# Plasticity in inhibitory networks improves pattern separation in early olfactory processing

**DOI:** 10.1101/2024.01.24.576675

**Published:** 2024-01-24

**Authors:** Shruti Joshi, Seth Haney, Zhenyu Wang, Fernando Locatelli, Brian Smith, Yu Cao, Maxim Bazhenov

**Affiliations:** 1 Department of Electrical and Computer Engineering, University of California San Diego, USA.; 2 Department of Medicine, University of California San Diego, USA.; 3 Department of Electrical, Computer and Energy Engineering, Arizona State University, USA.; 4 School of Life Science, Arizona State University, USA.; 5 Facultad de Ciencias Exactas y Naturales, Universidad de Buenos Aires, Instituto de Fisiología, Biología Molecular y Neurociencias, CONICET, Buenos Aires, Argentina.; 6 Department of Electrical and Computer Engineering, University of Minnesota, USA.

## Abstract

Distinguishing between nectar and non-nectar odors presents a challenge for animals due to shared compounds in complex mixtures, where changing ratios often signify differences in reward. Changes in nectar production throughout the day and potentially many times within a forager’s lifetime add to the complexity. The honeybee olfactory system, containing less than a 1000 of principal neurons in the early olfactory relay, the antennal lobe (AL), must learn to associate diverse volatile blends with rewards. We used a computational network model and live imaging of the honeybee’s AL to explore the neural mechanisms and functions of the AL plasticity. Our findings revealed that when trained with a set of rewarded and unrewarded odors, the AL inhibitory network suppresses shared chemical compounds while enhancing responses to distinct compounds. This results in improved pattern separation and a more concise and efficient neural code. Our Ca^**2+**^ imaging data support our model’s predictions. Furthermore, we applied these contrast enhancement principles to a Graph Convolutional Network (GCN) and found that similar mechanisms could enhance the performance of artificial neural networks. Our model provides insights into how plasticity at the inhibitory network level reshapes coding for efficient learning of complex odors.

## 1 Introduction

A honeybee can locate nectar-producing flowers by detecting floral aromas composed of many volatile compounds [1, 2]. However, nectar-producing and non-producing flowers contain many of the same compounds, making it difficult for the honeybee to determine the map between odor sensing and reward prediction [3]. This task is further complicated by the fact that nectar production may change within days and across locations, potentially many times within the foraging lifetime of a honey bee. Although the olfactory coding space is large (close to 1000 projection neurons in the honeybee antennal lobe, [4, 5]), mapping the sensory environment requires learning and relearning the association of reward with variable blends of volatile compounds. Nevertheless, honeybees can adapt to the variances in their environment owing to their keen ability to discriminate a wide range of olfactory stimuli [6–9].

Different forms of plasticity are a ubiquitous feature of the early olfactory processing in the brains of both mammals and insects. In the neural networks of the mammalian Olfactory Bulb (OB) and insect Antennal Lobe (AL), both nonassociative (unsupervised) and associative (supervised) forms of plasticity have been described [8, 10–12]. Together with the now well-established similarities in anatomical connectivity within the AL and OB networks [13], it is clear that early olfactory processing in these phylogenetically very different groups of animals works in much the same way to augment olfactory processing. Both associative and nonassociative plasticity have been shown to affect the AL and have been implicated in changes in odor representations [9, 14–18]. The rich repertoire of foraging behavior and a relative simplicity of olfactory network makes the honeybee an excellent model for investigating early olfactory plasticity.

In this new study, we combined Ca^2+^ imaging from honeybee AL with biophysically realistic computational modeling to characterize the role of inhibitory synaptic plasticity in changing odor representations after olfactory learning. We found that the representation of the rewarded odor changes to enhance contrast and thereby reduce the overlap between the representations of the rewarded and habituated odors in the AL. This change in representation, and hence an increase in discriminability, required both associative as well as nonassociative plasticity in the inhibitory synapses between AL neurons. Analysis of the Ca^2+^ imaging data revealed a similar change in odor representations after learning, supporting the model’s prediction. Applying these principles to an Artificial Neural Network (ANN) model trained to perform the odor categorization task revealed the mechanism of contrast enhancement for representations of the rewarded and habituated odors after training the model using backpropagation [19], a non-local and supervised approach. In sum, we demonstrate the role of inhibitory synaptic plasticity in the AL for effective odor discrimination and propose a novel computationally efficient mechanism for performing categorization tasks.

## 2 Results

### Differential conditioning created distinct representations of odors *in vivo*

**Fig. 1:**
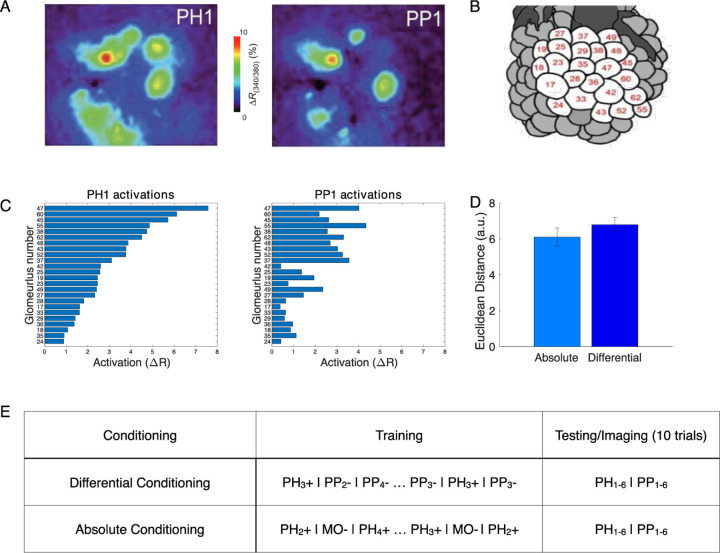
Olfactory representations in the honeybee AL. A) Calcium imaging responses elicited by two different odors (PH1 and PP1) in a representative bee. Here, PH1 and PP1 belong to different classes of odors, with PH odors being presented along with a reward (rewarded odors) and PP odors presented without any reward (habituated odors). B) Position of the glomeruli within the Antennal Lobe, for which Calcium Imaging responses were measured. C) Mean activation across the different glomeruli elicited by the PH1 and PP1 odors. Glomeruli are ordered with the glomeruli most activated by PH1 at the top and the glomeruli least activated by PH1 at the bottom. The same ordering is used for PP1 odor as well to highlight the difference in the activation patterns between the two odors. D) Euclidean Distance of glomeruli activation between different odor classes (PH and PP) increases after differential conditioning compared to absolute conditioning. This indicates that the glomerular representation gets more separable after differential conditioning. E) Honeybee training protocol for differential and absolute conditioning. In differential conditioning, the bee was trained with rewarded (PH) and habituated (PP) odors presented in a randomized order. For absolute conditioning, the bee was trained with only rewarded odors. Mineral Oil (MO) was presented to the bee without any reward during absolute conditioning. Each trial lasts for 4s, with 500ms of odor presentation (Data from [12]). The same training protocol was used for training the computational model.

In our previous work [12] we used Ca^2+^ imaging to test how representation of synthetic odor blends changes in the honeybee AL after differential conditioning (Figure 1A). Two synthetic classes of odors were based on varieties of the common snapdragon flower (*A. majus*): Potomac Pink (PP) and Pale Hybrid (PH) [20]. Each class was a blend of volatile chemicals that recapitulated mean and variance of naturally occurring PH and PP flowers. These two classes contained the same volatile chemicals at different ratios that makes distinguishing them a difficult task.

We found that odors within each class created distinct representations in the AL via spatial activation of different sets of glomeruli (Figure 1B,C). Further, these representations became more distinct after differential training. Specifically, in absolute conditioning, honeybees were rewarded after responding to PH odors; in differential conditioning honeybees were also habituated to the PP odors [12] (Figure 1E). We found that the distance between representations of PH and PP odors increased after differential conditioning (Figure 1D). Although the change in Euclidean Distance is not significant, the correlation between odor representations reduced significantly as shown in [12].

### Computational model of the mechanisms of differential conditioning

**Fig. 2:**
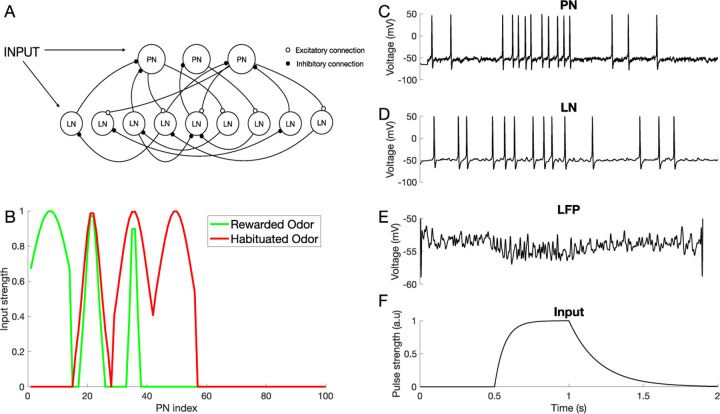
Computational Model of the Antennal Lobe. A) Architecture of the AL network Model (see Methods). B) Spatial pattern of odor stimuli for rewarded and habituated odors. The PNs are split into 7 groups, each corresponding to an odor percept. Percepts 1,2 and 3 are activated by the rewarded odor (green), while percepts 2,3 and 4 are activated by the habituated odor (red). C-D) Responses of a representative PN (C) and LN (D) to an odor input in the untrained network. E) Averaged PN activity (Local field potential). F) The 500ms time modulated current pulse which was provided as an inputs to individual neurons during odor stimulation (Gaussian noise was added to this input).

Here, we sought to investigate the mechanisms that produce differential learning between complex odors in the honeybee AL using a computational model. The model was a network of Hodgkin-Huxley type excitatory principal neurons (PNs) and inhibitory local neurons (LNs), representing honeybee AL network, and it was adapted from our previous work [16] (see Methods, Figure 2A).

To model rewarded learning - appetitive conditioning - we simulated activity-dependent presynaptic facilitation at both the LN-PN and LN-LN synapses (see Methods) inspired by the finding that octopamine receptors activated by reward signal are localized on the inhibitory LNs in the AL [21]. Octopamine is needed for appetitive olfactory learning [22], [7] and it has been shown to regulate inhibitory connections in the AL, affecting odor representations [23]. The activation of octopamine receptors in LNs together with odor induced activity causes a rise in *Ca*^2+^ that primes adenylyl cyclase to enhance cAMP and cAMP-dependent protein kinase activation that further regulates synaptic plasticity [24–27]. Therefore, we simulated the appetitive conditioning by increasing the synaptic weights of LN-PN and LN-LN synapses as the presynaptic LN was activated by the conditioned odor. Habituation was modeled as post-synaptic facilitation of LN-PN and LN-LN synapses, i.e., it depended on activity of postsynaptic LNs or PNs, based on the finding that habituation caused LN-PN facilitation in the fruit fly AL [14], [28].

To account for the fact that natural odors are composed of a blend of distinct volatile chemicals, we created inputs to the model combining activation of multiple distinct odor ‘percepts’. Each percept was comprised of a distinct population of ”virtual” ORNs that made connections with both LNs and PNs (mimicking the glomerular structure of the AL). This effectively divided all the LNs and PNs into several percept groups (Figure 2B). As with real floral odors, model odors were composed of multiple overlapping percepts creating a difficult discrimination task.

### Differential training expands the coding space in the maximally discriminatory dimension

**Fig. 3:**
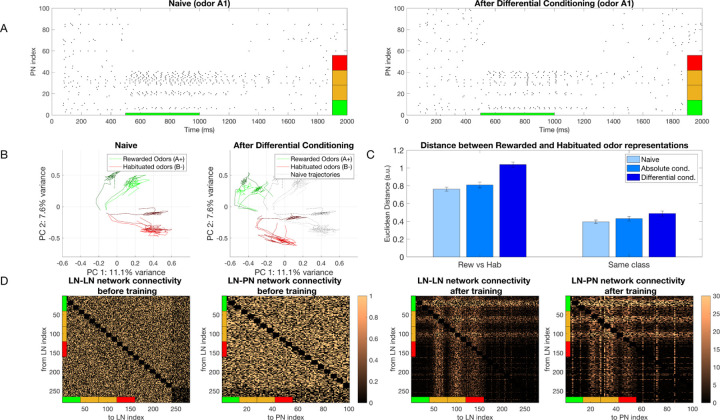
Change in representation of simple odors. A) Raster plot of the PN population response to rewarded odor (odor *A*_1_) before (left) and after (right) differential conditioning. Note, that activity of PNs activated by both rewarded and habituated odors (denoted by orange percepts) decreased while the activity of PNs activated uniquely by the rewarded odor (denoted by the green percept) increased. Red indicates percepts uniquely activated by the habituated odor. B) Dynamical trajectory of the spatiotemporal odor responses in two-dimensional (2D) PCA space. The green lines show trajectories for 3 Rewarded Odors and the red ones for 3 Habituated Odors. After training, the trajectories of the rewarded and habituated odors shift away from each other. Black lines on the right plot indicate naive trajectories. C) Left, the Euclidean Distance between the rewarded and habituated odors for naive network (light blue), and after absolute (middle tone blue) and differential (dark blue) conditioning. Right, the distance between odors from the same class. The Euclidean Distance is calculated for each pair of odors and then averaged over all the pairs of odors. The error bars show the standard deviation of the ED over different trials. D) Connectivity of the inhibitory networks before and after training. Connectivity of the inhibitory networks (LN-LN and LN-PN) after differential conditioning shows a grid-like structure which encodes the relationship between percepts belonging to the rewarded and habituated odors. The colorbars show the final value of the weight divided by its initial value.

We trained our model using differential conditioning, where one class of odors (*A*_1_*, A*_2_, …) was rewarded and another (*B*_1_*, B*_2_*, …*) was habituated. Within each class, different odors were simulated by changing the width of Gaussian for individual percepts (Figure 2B). The model network was exposed to a sequence of *N* = 30 odors total presented in a randomised order, for a total of 60s (each odor presentation lasted 2s). The plasticity rule for training was selected based on which type of odor (i.e., rewarded or habituated) was presented. We found that the training led to significant changes in the PNs’ population response for the rewarded odors (Figure 3A). Specifically, neurons associated with percepts that were unique to the rewarded odors showed enhanced firing rates after training, whereas neurons associated with percepts in the overlap between rewarded and habituated odors were strongly suppressed. For habituated odors, plastic changes led to rather minor and non-specific reduction of the PN responses. These combined effects effectively stretched the AL coding space used to represent odors.

We visualized effect of training using principal component analysis (PCA) (Figure 3B). We found that the trajectories of the naive response showed a relatively even distribution across PCA space. In contrast, after training, the sets of trajectories representing rewarded and habituated odors were strongly separated. We quantified this observation by measuring the time-averaged distance between odors. We found a significant increase in distance after differential training when comparing rewarded vs. habituated odors (p=1.9e-20) as well as when comparing odors within the same class, i.e., just rewarded or just habituated odors (p=1.88e-5) (Figure 3C, Supplementary Figure 2). All these changes to odor representation were due to changes in the inhibitory network, as these are the only synapses modified in the model (Figure 3D).

**Fig. 4:**
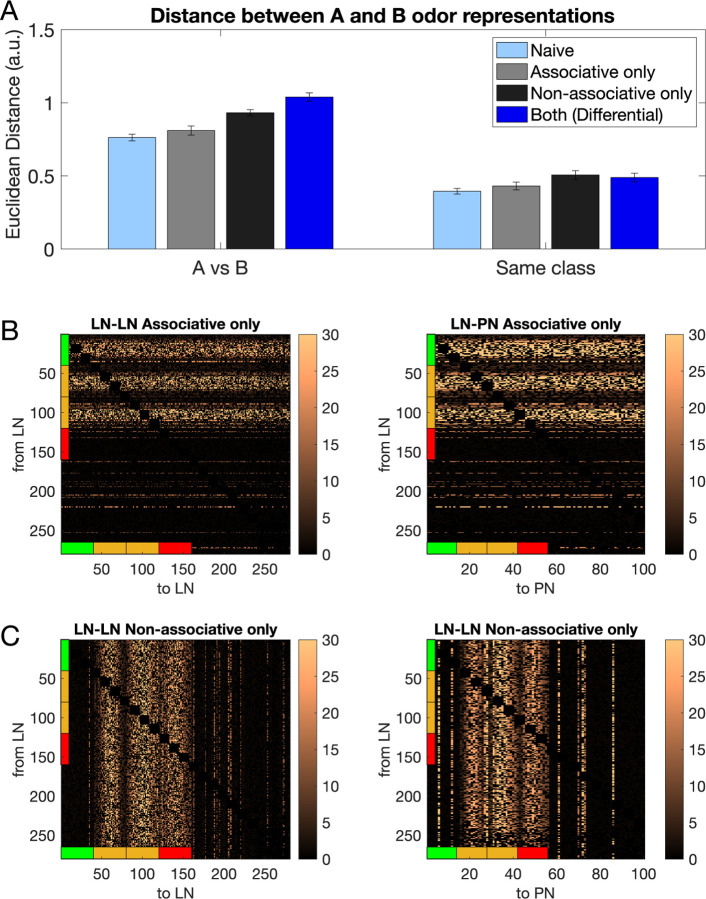
Connectivity of the inhibitory network after associative only and non-associative only training. A) Left shows the Euclidean Distance between rewarded and habituated odors increases after associative (light gray) as well as non-associative (dark gray) training compared to naive (light blue). The highest increase is seen when the bee is trained with both associative and non-associative training (dark blue). Right shows Euclidean Distance calculated for odors belonging to the same class. B) The LN-LN and LN-PN network after associative only training. C) The LN-LN and LN-PN network after non-associative only training. The colorbars show the value of the weight divided by its initial weight.

Next, we individually tested the effect of associative vs non-associative plasticity on the ability of the trained network to distinguish between the rewarded and habituated odors by selectively applying only pre or post-synaptic plasticity and measuring the distance between the learned odor representations. In Figure 4A, the Euclidean Distances between rewarded and habituated representations for different conditions - (i) Naive (pre-synaptic and post-synaptic plasticity are both off), (ii) Associative only (pre-synaptic plasticity is on, post-synaptic plasticity is off; absolute conditioning), (iii) Non-Associative only (pre-synaptic plasticity is off, post-synaptic plasticity is on) and (iv) Both (pre-synaptic and post-synaptic plasticity are both on; differential conditioning). We found that the largest increase in Euclidean distance is seen when both associative and non-associative plasticity are active (p=1.9e-20), followed by Non-associative only (p=1.5e-19) and Associative only (p=5.45e-18) in that order (Figure 4A, A vs B) when compared with the naive case. We found small but significant increases in the Euclidean Distance measured between odors of the same class for all 3 conditions compared to naive (Figure 4A, right, same class). The resulting learned LN-LN and LN-PN networks for the Associative only and Non-Associative only case can be seen in Figure 4B and C, respectively. Supplementary Figure 1 shows the raster plots for AL responses to a representative rewarded (A1) and a habituated odors (B1).

### Training with realistic odorants also produces contrast enhancement

**Fig. 5:**
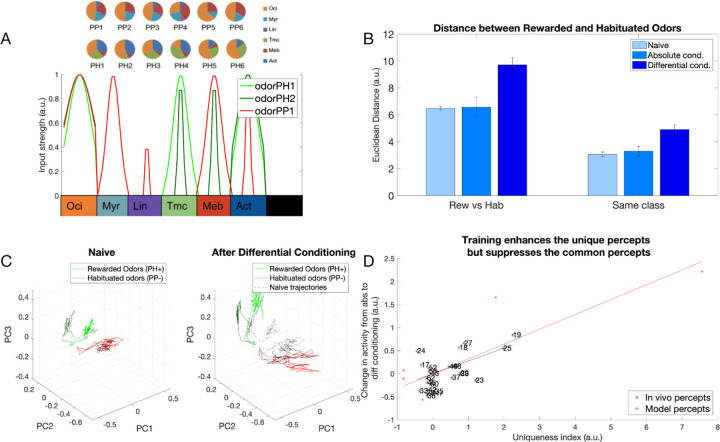
Contrast enhancement with complex odors based on in vivo data. A) Odors obtained from in vivo experiments (PH,PP) converted into inputs to the model (See Methods for details). B) Euclidean Distance between rewarded (PH) and habituated (PP) odors increases after differential conditioning (dark blue) compared to the naive network (light blue) and absolute conditioned network (middle tone blue). Distance within odors belonging to same class (e.g., PH1 vs PH2, PP1 vs PP2, etc) increases slightly after absolute conditioning (middle tone blue), similar to what is seen in experimental data, and significantly after differential conditioning (dark blue). C) The dynamical odor trajectory in the 3D PCA space shows the 3 rewarded (PH1, PH2, PH3) and 3 habituated odor (PP1, PP2, PP3) representations shifting in the direction which makes them more discriminable. D) Contrast enhancement based on Ca^2+^ imaging data (black) and model (red). After differential conditioning, activity in the glomeruli/percepts which have a high uniqueness index (i.e., uniquely activated by the rewarded odor) increases while activity in the glomeruli/percepts which have a low uniqueness index (i.e., activated by both rewarded and habituated odors) decreases (*R*^2^=0.3751, p=0.0015 for in vivo percepts and *R*^2^=0.7651, p=0.0225 for model percepts). Note, similar trend in vivo and in the model.

To ensure that the effects we have shown were not due to the specific construction of the model odors, we created a new set of odors where fractions of percepts were aligned with the fractions of the measured volatile chemicals present in each of the odor blend categories (i.e., PH and PP) that were used in behavioral experiments in [12]. These PH and PP odors were created by calculating the proportion of the chemical components present in the blends and setting the activation of each percept according to it (Figure 5A, see Methods for details). Training these odors using differential conditioning (PH was rewarded and PP was habituated) also produced a specific expansion of the coding space (Figure 5C); both the distance between odors of the different classes (i.e., rewarded and habituated, p=4.53e-21) and odors within the same class ((p=3.16e-15)) increased, in line with previous results. Another interesting observation was that the distance between odors belonging to the same class (PH1 vs PH2,… PP1 vs PP2,…) increases slightly after differential conditioning, reducing the similarity of the odor representations. Supplementary figure 2 shows the increase in distance between PH vs PH odors and PP vs PP odors. This finding has been also seen in vivo [12]. There, decrease in similarity of representations was found between different PH and PP odors, indicating that differential conditioning might make recognition of specific rewarded odors easier.

Inspired by our modeling results, where we found that representations of percepts that were unique to the odors were enhanced and representations of percepts that were common to many odors were suppressed leading to increase in Euclidean Distance between odors (Figure 5B), we sought to quantify this effect in vivo. Using the Ca^2+^ imaging data from honeybee (collected as described in [12]), we created a uniqueness score for each glomerulus (which is akin to our model percept). This was done by calculating for each glomerulus the difference between its activation by the rewarded odor and its activation by the habituated one (see Methods). We found a strong correlation between the uniqueness score of a glomerulus in the representation of an odor and the change in activity due to learning (Figure 5D). Specifically, glomeruli tended to increase their activity after training if they were unique in the representation of the rewarded odor and visa versa, in agreement with what we found in our model. This effect of expanding the coding space that aligns with the uniqueness of the percepts we term *contrast enhancement*.

### The inhibitory network adapts to discriminate between odors in a new environment via learning

As the honeybees move from one environment to another, the odors associated with reward and habituation may change. The animal needs to learn the association of these new odors with the reward contexts present in the new environment. The inhibitory network, hence, must be flexible enough to be able to learn representations for the new odors and be able to successfully discriminate between them. In order to test whether the network can adapt to a new environment, we constructed two non overlapping sets of odors (P/Q - environment 1 and S/T - environment 2). All PNs and LNs were divided into 8 percept groups with each odor activating 3 percepts. Odor class P activated percepts 1,2,3; odor class Q activated percepts 2,3,4; odor class S activated percepts 5,6,7; and odor class T activated percepts 6,7,8. Thus, the odor classes from the 2 environments had no overlap, but the odor classes within an environment had an overlap of 2 percepts. In the first environment, Env1, on which the network was trained, odor P was associated with reward (P+) and odor Q was associated with habituation (Q-). In the second environment, Env2, odor S was associated with reward (S+) and odor T was associated with habituation (T-). We studied effect of sequential training of odors from the different environments in two settings: 1) when there was no delay between training the 1^*st*^ and the 2^*nd*^ environment, and 2) when there was a large delay between training the 1^*st*^ and the 2^*nd*^ environment. Testing was done by exposing the network to all the odors (P,Q,S,T) after training in each environment. To simulate unlearning, any plastic changes acquired by the model decayed with the time constant 30 sec in absence of spiking activity (see Methods).

**Fig. 6:**
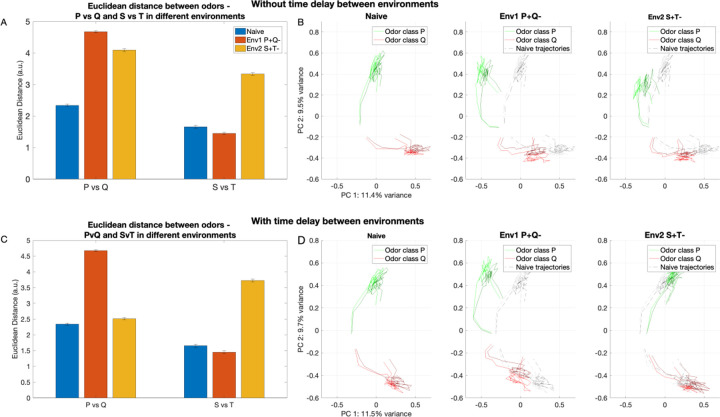
Learning by AL inhibitory network in two different environments. Environment 1 (Env1): P+Q-; environment 2 (Env2): S+T-. The top panels (A,B) show the condition where the model is exposed to the second environment immediately after the first environment. The bottom panels (C,D) show the condition where the model is exposed to the second environment after learning from the first environment has decayed. A) Euclidean distance between odor classes P and Q (first group of bars) increases when the model is trained on Env1 (P+Q-, red bars) (p=2.9e-12). The Euclidean distance between odor classes S and T shows no significant change (second group of bars, p=0.1). When the model is further trained on Env2 (S+T-, yellow bars) immediately after Env1, the distance between P and Q remains high while the distance between S and T increases significantly (p=1.9e-11). This shows that the network learns the relationship between percepts from Env2 while retaining memory of the relationship between percepts learned in Env1. B) The dynamical trajectories of representations of odor classes P (green) and Q (red) shown in different environments: naive, Env1 (P+Q-) and Env2 (S+T-). Here, we see the representations of P and Q shift away from one another when the model is trained in Env1 and remain that way when there is no time delay between training Env1 and Env2. C) Same as in (A) but Env2 was trained after delay. The time delay between training Env1 and Env2 allows for the plastic changes formed after Env1 training to decay, resulting in the Euclidean distance to reduce back to naive level after training on Env2, so the network only learns the relationship between percepts from Env2. D) Same as B. Here, we see the representations of P and Q shift away from one another when the model is trained on Env1 but returns back to its original (naive) trajectories after training on S+T- since the large time delay allows for the weights in the model representing the relationship between percepts of odors P and Q to decay back to their baseline values.

As expected, the Euclidean Distance between the representations of odors P and Q increased after the network was trained in the first environment (P+Q-), while the distance between odors S and T did not change (Figure 6A). When network was trained subsequently on the second environment (S+T-) without delay, the distance between P and Q remained high, while the distance between S and T increased indicating that the network retained memory of the first environment while also learning the odor representations in the second environment (Figure 6A). This can also be seen in the PCA trajectories of odors P and Q tested after each training stage. As seen in Figure 6B, the separation between odor P and Q increased after training on Env1 and the rewarded odor (P) did not return to the naive trajectory when tested after training on Env2, if there was no delay before Env2 training. In contrast, when there was a long time delay between training in the Env1 and Env2, the distance between odors P and Q increased after training on Env1 but decreased again when tested after training on Env2 (Figure 6C). The PCA trajectory of the rewarded odor (P) can be seen deviating away from the habituated odor (Q) after training in Env1 but returns close to the original (naive) trajectory later (Figure 6D). This suggests that with a long enough time delay between training in the two environments, the network only learns the relationship between percepts from the most recent environment (Env2) and forgets the odor representations learned after the first environment.

Together, these results indicate that forgetting in our model results from an independent active process (synaptic weight decay) and not from interference between memories, and that the learning different environments is accomplished by independent subsets of inhibitory weights in the AL network.

### Graph convolution neural networks and inhibitory learning converge on similar solutions

Can the principles learned from olfaction be exploited to improve performance of artificial neural networks (ANNs)? The encoding of odors in the AL takes place by learning the relationships between the components of the odors and the reward associated with the odors. Graph convolutional network (GCN) is a class of ANNs that take graph data as the input and aggregates information from neighboring nodes of the graph to perform a task (e.g., classification) [30, 31]. This enables the GCN to utilize the relationships between nodes to improve the classification performance [31]. Hence, GCNs can be considered analogous to the AL, in that they encode relationships between different components of the input and utilize these relationships to categorize the inputs.

**Fig. 7:**
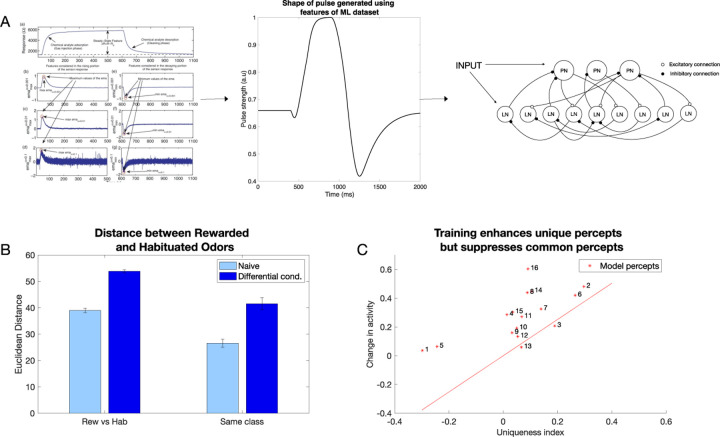
Contrast enhancement in larger network model with input generated using chemical gas sensor data (Reproduced from [29]). A) The features extracted from the response curves are converted into a pulse that is then fed into the larger biophysical model as an input. B) Euclidean distance between and within classes increases after differential conditioning training. C) Contrast enhancement analysis in the large biophysical network with Gas Sensor Array Drift Dataset inputs shows similar results to the smaller biophysical model and in vivo data (*R*^2^ = 0.416, p=0.007)(compare to Fig. 5D).

**Fig. 8:**
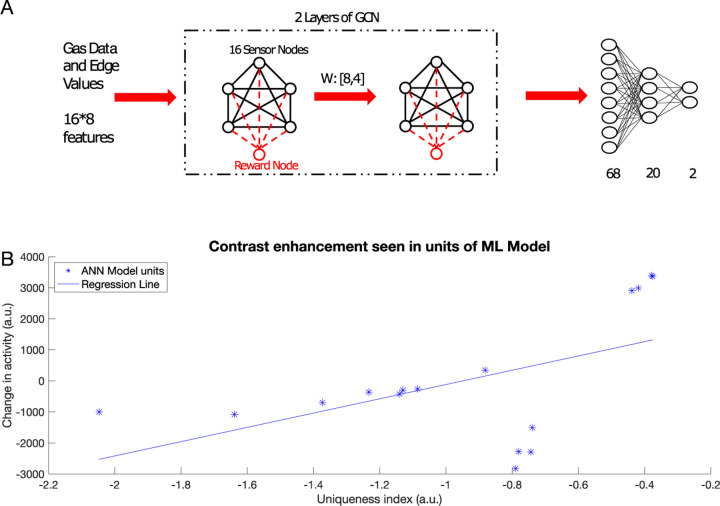
Contrast enhancement in a machine learning model. A) Schematic of the GCN model. The model receives features from the gas sensor array drift data set and processes it through two graph convolutional layers. The graph is then unrolled into a vector and sent through 2 fully connected layers for categorization. Training weights between GCN layers can be thought of as analogous to the AL biophysical model training. B) Change in activity in the graph nodes in the first and second layer of the GCN vs uniqueness index of that node (*R*^2^ = 0.276, p=0.031). Note contrast enhancement: nodes with higher uniqueness tend to increase the activity and those with lower uniqueness index tend to decrease the activity.

Here, we compared results obtained using our biophysical model of the honeybee AL network to a GCN. In order to do that, we trained each network on chemical gas sensor data [29], where the biophysical network model was trained via the inhibitory plasticity (as described before) (Figure 7) and the GCN was trained using backpropagation (see Methods) (Figure 8). The numeric features in this dataset, which included response magnitude, On and Off time constants, and others (see Methods), were used directly to train the GCN model. In the biophysical model, we used three features (response magnitude and On and Off time constants) from the data to construct input pulses to model odor stimulation (Figure 7A).

We selected half of the odors to be associated with the reward and the other half to be habituated. Learning in the biophysical model increased the discriminability between the rewarded and habituated odors as indicated by the increase in Euclidean Distance (Figure 7B).

The GCN network (Figure 8A) was able to identify which class an odor belonged to (i.e., rewarded or habituated) with 88% accuracy. We found that both the GCN and the biophysical model showed the same characteristics in the changes to representations of odors. Specifically, units that represented components that were unique to an odor were enhanced and units that represented components common to many odors were suppressed (Figure 8B). Both the top-down approach, rooted in backpropagation, and the bottom-up approach, grounded in local biological learning rules, converge to the same contrast enhancement strategy. This convergence highlights its effectiveness as a computational strategy for accomplishing categorization tasks.

## 3 Discussion

In vivo recordings from the honeybee’s antennal lobe (AL) have found that separation among patterns of the neural activity representing odor blends from different varieties of flowers improves after differential reinforcement for each variety [12]. However, the mechanisms behind this phenomenon remain poorly understood. The primary goal of this new study was to reveal plasticity mechanisms leading to modification of the neural representations of the natural odor blends in the early olfactory system - honeybee AL - using a combination of computational modeling, machine learning and Ca^2+^ imaging.

Natural odors are generally blends of several chemical components [1]. These odors are present in different concentrations, and nectar production may also change from season to season and environment to environment. Although the olfactory coding space is large, optimally mapping the sensory environment requires learning and relearning the association of reward with variable blends of volatile compounds [1]. We found that, through a combination of associative and non-associative plasticity in the inhibitory AL network, the common components in the neuronal representations of the rewarded and habituated odors decreased their activity, while the component unique to rewarded odors increased their activity. This led to a decrease in overlap between the representations of the two odor classes, while also making the representation of the rewarded odors more compact. This ”contrast enhancement” effect was also observed in the Ca^2+^ imaging data, consistent with the computational model. When subsequently stimulated by odors from a different environment, the inhibitory network learned the odor-reward associations from the most recent environment, demonstrating the adaptability of the inhibitory network to changes in the olfactory environment. To test the application of these principles to Artificial Intelligence, we developed and trained a machine learning model based on the Graph Convolutional Network (GCN) that performed the same task of categorizing complex odors. Analyzing the activity of the units in the GCN, we found a similar strategy of changes in the representation of the rewarded odors as observed in the biophysical model and in vivo data.

Previous studies have successfully applied computational network models to study mechanisms of odor-triggered oscillations [32–36] and odor-induced plasticity [9, 16, 37–41] in the early olfacory system of insects. The model in [16] revealed that stimulus-specific changes in synaptic inhibition are sufficient to explain shifts in odor representations after olfactory learning. The study by [9] used both firing rate and conductance-based models to explore non-associative plasticity in the AL. Building upon these earlier works, we developed the honeybee AL model with inhibitory synaptic plasticity and tested it in response to different odor classes denoting different varieties of floral odors, with a specific amount of overlap between them. Our results demonstrate that a combination of associative and non-associative plasticity within the inhibitory AL network plays a crucial role in improving odor separation. These results can be generalized beyond insect olfaction. The main principles of nonassociative and associative plasticity operating in the first relay for olfactory coding are similar in the brains of insects and vertebrates [8–12].

Biogenic amines have been shown to play an important role in reinforcement during olfactory learning [22, 42, 43]. In the honeybee, a cluster of cells at the base of the brain called Ventral Unpaired Medial (VUM) cells receive input from sucrose (US)-sensitive taste receptors [22, 44–47]. The outputs from VUM cells spread broadly throughout association areas of the brain, including the AL and the MB, where they release the biogenic amines octopamine and tyramine as the reinforcement signal. Immunological studies have shown that the octopamine receptor (AmOA1) is expressed alongside GABA receptors in the honeybee AL [21]. Other studies [23] have shown that octopamine modulates odor representations in PNs by regulating inhibitory connections in the AL. Repeated or prolonged exposure to a stimulus without any reinforcement reduces the behavioral response to those odors; this is called habituation [38, 48, 49] and leads to phenomenon of latent inhibition [49, 50]. Studies in the fruit fly AL have revealed that habituation arises from the facilitation of inhibitory LN to PN synapses involved in odor representation [14, 28].

Honeybees have a rich repertoire of learning behaviors to odors that range from nonassociative through associative and operant conditioning [3, 51]. Behavioral studies revealed that differential conditioning enables honeybees to discriminate between rewarded and habituated odors [12]. Study [52] found that the AL inhibitory network plays an important role in modifying projection neuron (PNs) responses after learning. It has been reported that the network effect of plasticity in the honey bee AL is to ‘warp’ the coding space around odors that are relevant to solving a current problem, such as identifying food [12, 53]. To reveal the mechanism behind this dynamics, we analyzed PN responses and found that the neuronal component in the representation of the rewarded odor that was unique to that odor was enhanced, while the components that had an overlap with the other odor class (habituated odor class) were suppressed. This increased the contrast and, hence, the separation between the odor classes. Model predictions were confirmed by Ca^2+^ imaging data analysis, which revealed that honeybee glomeruli representing common components of odors were suppressed, while components unique to the rewarded odor were enhanced after learning. Agreeing with this result, imaging studies in the honeybee [12, 52, 54, 55] and other insects [56] have shown that odor representations in the AL change after differential conditioning, with rewarded and unrewarded odor representations becoming less correlated. These findings support the notion that the increase in the separation of odor representations in the AL in vivo occurred through contrast enhancement, thus validating the prediction made by our model.

We found that the model needs to implement both associative and non-associative plasticity to explain in vivo data. The suppression of the common component in the rewarded odors was driven by an increase in the inhibitory LN to PN connection strength, caused by postsynaptic (non-associative) plasticity. This increase resulted from the firing of PNs triggered by the habituated odor, aligning with the findings of [9] and [16]. These studies demonstrated that non-associative inhibitory plasticity from LNs to PNs played a crucial role in ‘filtering out’ components of the habituated odor after honeybee was exposed to the odor without receiving a reward. The increase in the activity of the unique component in the rewarded odors occurred due to both presynaptic and postsynaptic facilitation, leading to increase in inhibition of the LNs that were inhibiting PNs representing the unique component (i.e., PNs’ disinhibition). A similar effect, an increase in the activity of the glomeruli for the rewarded but not the habituated odor, was also observed in [54]. Together, these two mechanisms contributed to shifting the PN responses of the rewarded odor away from the habituated odor. We also observed that, in general, this effect was specific to the most recent odor environment, and changing the odor environment could lead to relearning thereby demonstrating the efficiency of the adaptive mechanisms represented by the plastic inhibitory AL network.

The Mushroom Body (MB), the next layer in olfactory processing in insects, is considered a major center for associative learning and olfactory memory [42]. Information from the PNs in the AL is relayed to the MB, where Kenyon Cells (KCs) represent odors with a sparse firing patterns [57, 58]. Remarkably, approximately 800 PNs synapse onto around 170,000 KCs [59, 60], significantly increasing the dimensionality of the odor representation space. Experimental evidence has shown that KC responses are influenced by associative reinforcement learning, which stabilizes odor representations in KCs. In contrast, odor presentations without any reinforcement weaken their representation in KCs [61]. Modeling studies [62] have also indicated that the categorization of odors in the MB depends on changes in PN responses caused by conditioning. Our study suggests a potential mechanism for this change in KC responses. The rewarded odor triggers more population-specific AL activity after training, and the KCs connected to these PNs display more reliable firing after training since they receive stronger activation from the PNs, as suggested by [61].

Artificial Intelligence has made rapid progress in recent years in solving tasks such as image classification, speech recognition, natural language processing. Although it has now grown into a separate field, AI can trace its roots back to neuroscience with the earliest artificial neural networks trying to mimic information processing in the brain [63]. Currently, efforts are being made to incorporate biological principles to develop new generation of AI. Graph Neural Networks (GNNs) have been developed to work with graph data. There have been significant advances in GNNs, increasing their capabilities and expressive power [31], [64]. In one study GNNs have been used to learn a generalizable perceptual representation of odors [65]. Here, we developed a Graph Convolutional Network model (GCN) as an artificial parallel to the insect Antennal Lobe. We trained this network to perform an odor categorization task and analyzed the activity of the artificial neurons in the first and second layers of the GCN. This analysis showed that the contrast between representations of different odor classes increased based on the same mechanism described for the biophysical model. Together, these results suggest that contrast enhancement is an efficient strategy for performing a categorization task.

In summary, our study has unveiled the circuit-level mechanisms of olfactory learning in the honeybee AL, enhancing odor discrimination. This learning paradigm relies on inhibitory network plasticity, which enables the reshaping of the coding space to align with the current task and environment. These findings propose an effective computational strategy for the perceptual learning of intricate natural odors, achieved through the modification of the inhibitory network during early sensory processing.

## 4 Methods and Materials

### Computational Modeling

We constructed a biophysical model of the honeybee Antennal Lobe (AL), containing 100 excitatory Projection Neurons (PNs) and 280 inhibitory Local Interneurons (LNs). Each PN and LN was modeled by a single compartment that included voltage- and calcium-dependent currents described by Hodgkin-Huxley kinetics [66]. The model here was adapted from our previous studies [67], [16] and is available for download from the Bazhenov lab website. Model equations were solved using a fourth order Runge-Kutta method with an integration time step of 0.04ms.

### Membrane Potentials

PN and LN membrane potential equations [66] are given by

$$C_{m}\frac{dV_{PN}}{dt}=-g_{L}\left(V_{PN}-E_{L}\right)-I_{Na}-I_{K}-I_{A}-I_{T}-I_{h}-g_{KL}\left(V_{PN}-E_{KL}\right)-I_{GABA_{A}}-I_{nACh}-I_{stim}$$

$$C_{m}\frac{dV_{LN}}{dt}=-gL\left(V_{LN}-E_{L}\right)-I_{N\alpha }-I_{K}-I_{T}-g_{KL}\left(V_{LN}-E_{KL}\right)-I_{GABA_{A}}-I_{nACh}-I_{stim}$$

The passive parameters are as follows: For PNs, $C_{m}=2.9*10^{-4}\mu F$, *g*_*L*_ = 0.01*mS/cm*^2^, *g*_*KL*_ = 0.012*mS/cm*^2^*, E*_*L*_ = −70*mV, E*_*KL*_ = −95*mV*.

For LNs, *C*_*m*_ = 1.43 ∗ 10^*−*4^*µF*, *g*_*L*_ = 0.05*mS/cm*^2^, *g*_*KL*_ = 0.018*mS/cm*^2^*, E*_*L*_ = −70*mV, E*_*KL*_ = −95*mV*. An external DC input was introduced to each neuron through *I*_*stim*_.

The intrinsic currents, including a fast sodium current, *I*_*Na*_, a fast potassium current, *I*_*K*_, a transient potassium A-current, *I*_*A*_, a low threshold transient *Ca*^2+^ current, *I*_*T*_, a hyperpolarization activated cation current *I*_*h*_ are given by equation

$$I=gm^{M}h^{N}\left(V-E\right)$$

where the maximal conductances for PNs are *g*_*Na*_ = 90*mS/cm*^2^*, g*_*K*_ = 10*mS/cm*2*, g*_*A*_ = 10*mS/cm*^2^*, g*_*T*_ = 2*mS/cm*^2^*, g*_*h*_ = 0.02*mS/cm*^2^. The maximal conductances for LNs are *g*_*Na*_ = 100*mS/cm*2*, g*_*K*_ = 10*mS/cm*^2^, and *g*_*T*_ = 1.75*mS/cm*^2^.

For all cells, *E*_*Na*_ = 50*mV, E*_*K*_ = 95*mV,* and *E*_*Ca*_ = 140*mV*. The gating variables 0 ≤ *m*(*t*)*, h*(*t*) ≤ 1 satisfy the following:

$$\frac{dm}{dt}=\frac{m_{\infty }\left(V\right)-m}{\tau_{m}\left(V\right)}$$

$$\frac{dh}{dt}=\frac{h_{\infty }\left(V\right)-h}{\tau_{h}\left(V\right)}$$

where the steady state (e.g., *m*_*∞*_(*V* )) and relaxation times (e.g., *τ*_*h*_(*V* )) are derived from experimental recordings of the specific ionic currents. These distinct voltage-dependent functions are given in [16]. For all cells, intracellular *Ca*^2+^ dynamics were described by a simple first-order model as follows:

$$\frac{d\left[Ca^{2+}\right]}{dt}=-A\cdot I_{T}-\frac{\left[Ca^{2+}\right]-{\left[Ca^{2+}\right]}_{\infty }}{\tau }$$

where [*Ca*^2+^]_*∞*_ = 2.4 · 10^*−*4^*mM* is the equilibrium of intracellular *Ca*^2+^ concentration, *A* = 5.2 · 10^*−*5^*mM* · *cm*^2^*/*(*ms* · *µA*) and *τ* = 5*ms*.

### Synaptic Currents

Fast GABA and cholinergic synaptic currents to LNs and PNs were modeled by first-order activation schemes [68]. Fast GABA and cholinergic synaptic currents are given by:

$$I_{syn}=g_{syn}\left[O\right]\left(V-E_{syn}\right)$$

where the reversal potential is *E*_*nACh*_ = 0*mV* for cholinergic receptors and *E*_*GABA*_ = −70*mV* for fast GABA receptors. The fraction of open channels, [*O*], is calculated according to the equation

$$\frac{d\left[O\right]}{dt}=\alpha \left(1-\left[O\right]\right)\left[T\right]-\beta \left[O\right]$$

For cholinergic synapses transmitter concentration, [*T* ], is given by

$$\left[T\right]=A\cdot H\left(t_{0}+t_{\text{max}}-t\right)\cdot H\left(t-t_{0}\right)$$

and for GABAergic synapses

$$\left[T\right]=\frac{1}{1+\text{exp}\left(-\left(V-V_{0}\right)/\sigma \right)},$$

where *H* is the Heaviside step function, *t*_0_ is the time of receptor activation, *A* = 0.5*, t*_*max*_ = 0.3*ms, V*_0_ = 20*mV*, and *σ* = 1.5. The rate constants were given *α* = 10*ms*^*−*1^ and *β* = 0.2*ms*^*−*1^ for GABA synapses and *α* = 1*ms*^*−*1^ and *β* = 0.2*ms*^*−*1^ for cholinergic synapses. The peak synaptic conductances were set to *g*_*GABA*_ = 0.02*µS* between LNs, *g*_*GABA*_ = 0.015*µS* from LNs to PNs and *g*_*ACh*_ = 0.3*µS* from PNs to LNs.

For simulating the larger network with Gas Sensor Array Drift Dataset as input, the peak synaptic conductances were as follows: *g*_*GABA*_ = 0.024*µS* between LNs, *g*_*GABA*_ = 0.019*µS* from LNs to PNs and *g*_*ACh*_ = 0.075*µS* from PNs to LNs.

### Plasticity

A simple phenomenological model of synaptic facilitation was used to model inhibitory plasticity (from LN to LN and LN to PN). Specifically, the inhibitory network underwent presynaptic facilitation when presented with a rewarded odor and postsynaptic facilitation when presented with a habituated odor during learning [16]. Presynaptic (associated with reward) facilitation was assumed to depend on the octopamine receptors AmOA1 expressed in inhibitory LNs in the honey bee AL [21], [69] and it was based on the spiking events of the presynaptic neurons. In contrast, postsynaptic facilitation (associated with odor habituation) was based solely on the spiking event of the postsynaptic neuron [14], [18].

To model facilitation, the maximum synaptic conductance is multiplied by a facilitation variable, *F*. *F* is updated each time there is a spike:

$$F=F_{i}+dF_{pre}+dF_{post}$$

where *dF* is facilitation rate (*dF*_*pre*_ = 0.15 and *dF*_*post*_ = 0 for presynaptic facilitation and *dF*_*pre*_ = 0 and *dF*_*post*_ = 0.15 for postsynaptic facilitation). *F*_*i*_ is the value of the facilitation variable just before the *i*^*th*^ spike. In the absence of any spiking events, *F* had an exponential decay according to the following equation

$$F_{t}=1+\left(F_{t-1}-1\right)\text{*exp}\frac{-\left(t-t_{i}\right)}{\tau }$$

In the above equation, *τ* = 30s is the time constant of the decay; (*t* – *t*_*i*_) is the time since the *i*^*th*^ spiking event; *F*_*t*_ corresponds to the value of F at time *t* with the initial value *F*_0_ = 1. The time constant of decay is the same for pre and postsynaptic plasticity. The updated synaptic weights at the end of the training period (30 presentations, 60,000s) were frozen and used as the synaptic weights in the testing phase.

### Network Geometry

The AL network included 100 PNs and 280 LNs. All these PNs and LNs were organised into 20 glomeruli, with each glomerulus containing 12 LNs (unipolar LNs) and 5 PNs. In addition to these, there are 40 LNs that contribute to global inhibition (multipolar LNs). The connections between each group of neurons were generated randomly based on the probabilities described in Table 1

In order to accommodate the ML-dataset inputs to the model, in some simulations we increased the number of neurons to 400 PNs and 1120 LNs. The larger network has random connectivity between LNs, LNs to PNs and PNs to LNs with a probability of 0.125. There were no connections between the different PNs.

### Odor Stimulation

To model odor stimulation, a selected fraction of LNs and PNs was activated by a current pulse with a rise time constant of 66.7ms and a decay time constant of 200ms for a period of 500ms as can be seen in Figure 2F. Small amplitude currents in the form of Gaussian Noise were added to each cell, to ensure random and independent membrane potential fluctuations. The total network of 100 PNs and 280 LNs was separated into 7 smaller, uniform groups of cells. Next, we defined the smallest unit of an odor input - a percept, each percept activated one such group of PNs and LNs. For each odor, 3 out of 7 available percepts were assigned. Thus, odor classes were defined based on which 3 out of the 7 percepts were active for a given class. Individual odors within a class were defined by changing the level of LN/PN activation triggered by percepts. The odors were divided into two classes – one class associated with reward and another with no reinforcement, with an overlap of 2 percepts.

In some simulations we modeled ”PH-PP” odors which were created from the chemical compositions of two varieties of snapdragon odor blends (flowers) – PH (Pale Hybrid) and PP (Potomac Pink) as used in [12]. Since there are 6 chemical components present in each odor, each chemical component was assigned a percept and the level of activation of each percept was calculated based on the proportion of that chemical component present in the natural odor blend. The 7^*th*^ percept remained inactive for all odors. For example, in the odor PH1, Oci chemical is 37.2% of the mixture. Hence, the standard deviation for the Gaussian for percept 1 is set to 0.372 (Figure 5A).

Finally, we also used odor classes derived from the UCI Gas sensor dataset [29]. This dataset consists of data for 6 odors, obtained from 16 metal oxide sensors. The set of 400 PNs and 1120 LNs was divided into 32 groups, each group activated by a percept. The first 16 groups were provided with odor pulses, created from 3 features of the data from each metal oxide sensor - ∆*R*, *ema_max*_*α*=0.001_ and *ema_min*_*α*=0.001_ [29]. From these 3 features, we created an odor pulse with a rise time constant proportional to 1*/ema_max*_*α*=0.001_, decay time constant proportional to 1*/ema_min*_*α*=0.001_ and the maximum height of the pulse proportional to ∆*R*. The remaining 16 percepts were left inactive, in order to maintain the E/I balance of the network. Of the 6 odors, 3 were associated with reward and 3 were associated with no reinforcement.

### Machine Learning Model - GCN model

In the last section of this paper, we applied a Graph Convolutional Network (GCN), a type of Graph Neural Network (GNN), to simulate AL training using machine learning approach. GCN is an approach for semi-supervised learning on graph structured data. It is convolutional in nature, because filter parameters are typically shared over all locations in the graph. GCN is a generalized version of CNNs which operate directly on arbitrarily structured graphs [30]. In [31], the choice of convolutional architecture is motivated via a localized first-order approximation of spectral graph convolutions.

For the GCN model, the goal is to learn a function of signals/features on a graph which takes as input:

- A feature description *x*_*i*_ for every node *i*; summarized in a N×D feature matrix *X* (N: number of nodes, D: number of input features)
- A representative description of the graph structure in matrix form; typically in the form of an adjacency matrix *A* (or some function thereof) and produces a node-level output *Z* (an N×F feature matrix, where F is the number of output features per node). Graph-level outputs can be modeled by introducing some form of pooling operation. [30]

Every neural network layer can then be written as a non-linear function *H*(*l* +1) = *f* (*H*(*l*)*, A*) with *H*(0) = *X* and *H*(*L*) = *Z*, *L* being the number of layers. The specific models then differ only in how *f* is chosen and parameterized.

The UCI Gas Sensor Array Drift Dataset was taken as the input to the GCN, which consists of 2 parts, i.e, the graph convolutional layers and the fully connected layers. This dataset contains data for 6 different odors from 16 metal oxide sensors, with 8 features extracted from each sensor response, as described above. Thus, each odor is made of 128 features from 16 sensors. The data was normalized by subtracting the mean and dividing by standard deviation for each sensor value over the training and test sets. The GCN model consists of 16 (sensor) + 1 (reward/habituation) nodes and 2 layers of graph convolution. The output of the GCN is converted to a vector and sent to 2 fully connected layers for the final classification. The 6 odors from the dataset are split into 2 groups, one group associated with reward and one group associated with habituation. The task being performed by the model is to determine which group the given odor belongs to. The equation of this GCN model is, then, *H*(*l* + 1) = *h*(*A H*(*l*) *W* (*l*)), where *l* is the current layer and *A* is the graph adjacency matrix with all connections between nodes belonging to sensors set to 1. The reward/habituation nodes’ connections to the main 16 node graph was set during training as follows: if a rewarded odor was presented to the model during training, the connections between the reward and sensor nodes was set to 1. Conversely, if a habituated odor was presented to the model during training, the connection between reward node and sensor nodes was set to −1.

The model was trained via backpropagation, with the matrix W and the fully connected head of the model being updated during training. The cross entropy loss was optimized with the Adam Optimizer with a learning rate of 0.01 for 20 epochs. The graph convolution part of the model takes in 8 features from the 16 sensors and transform them into 4 abstract ones at the second layer. These abstract features encode the relationships between the different sensor features during training, similar to the AL which learn the relationships between the different odor percepts during training. Hence, we can say that the GCN model performs input preprocessing by learning the relationships between input features similar to the AL in honeybees. The first layer of the GCN can be thought of as analogous to the untrained AL network and the second layer can be thought of as the trained AL network.

### Analysis

#### Euclidean Distance

Euclidean Distance (ED) was calculated based on the normalized binned spike counts across all the PNs for a single trial during the period of stimulation, with a bin size of 100ms. Normalization of the 100 dimensional vector for each time bin was done by dividing by the norm of the vector. The ED was calculated for each time bin and each trial and averaged over them.

#### PCA trajectory

The response trajectory of the entire PN population to a specific input was constructed by binning the spike trains in 40ms bins. For visualisation, the 100 dimensional space of PN responses was reduced to 2D/3D space using Principal Component Analysis (PCA).

#### Uniqueness index calculation

The uniqueness index for each glomerulus/percept was calculated as follows:

$$\text{UI}=\frac{\left(\;act\_glom{\;}_{naive}^{rew}-\;act\_glom{\;}_{naive}^{hab}\right)}{\;act\_glom{\;}_{naive}^{hab}}$$

and the change in activity of the neurons:

$$\text{Change in activity }=\frac{\;act\_glom{\;}_{diff}^{rew}-\;act\_glom{\;}_{naive}^{rew}}{\;act\_glom{\;}_{naive}^{rew}}$$

where *act* $act\_glom{\;}_{naive}^{rew}$ denotes the activation of the glomerulus by the rewarded odor and $act\_glom{\;}_{naive}^{hab}$ denotes the activation by the habituated odor before training (in the naive condition) and $act\_glom{\;}_{diff}^{rew}$ denotes the activation of the glomerulus by the rewarded odor after differential conditioning. A high positive Uniqueness index (UI) indicates that the given glomerulus is uniquely activated by the rewarded odors and a UI close to 0 would indicate the glomerulus is activated to a common extent by both rewarded and habituated odors. This method of calculating the UI was used for the biophysical models.

The uniqueness index for the GCN model was calculated based on the average activation (average of 8 feature values) for each sensor in GCN layer 1 for rewarded and habituated odors. The UI for the GCN is:

$$\text{UI}=\frac{\;act\_unit_{L1}^{rew}-act_{-}unit_{L1}^{hab}}{act_{-}unit_{L1}^{hab}}$$

where $act\_unit_{L1}^{rew}$ corresponds to the average activation of the GCN unit for the rewarded odors in layer 1 and $act_{-}unit_{L1}^{hab}$ corresponds to the average activation of the GCN unit for the habituated odors in layer 1. The change in activity was calculated based on the difference in average activation of each sensor node between layer 1 and layer 2 of the GCN:

$$\text{Change}\;\;\text{in}\;\;\text{activity}\;=act\_unit_{L2}^{rew}-\;act\_unit_{L1}^{rew}$$

## Supplementary Material

Supplement 1

## Figures and Tables

**Table 1:** Connection probabilities between different neuron groups in the AL.

Source Unit	Target Unit	Connection Probability
All PNs	All PNs	0
	Unipolar LNs	0.4
	Multipolar LNs	0.4

	PNs from same glomerulus	0
Unipolar LNs	PNs from different glomerulus	0.5
	Unipolar LNs from same glomerulus	0
	Unipolar LNs from different glomerulus	0.4
	Multipolar LNs	0.3

Multipolar LNs	All PNs	0.3
	Mutlipolar LNs	0.1
	Unipolar LNs	0.4

## Data Availability

The data will be made available after publication.
