# Plasticity in inhibitory networks improves pattern separation in early olfactory processing

**DOI:** 10.1101/2024.01.24.576675

**Published:** 2024-07-25

**Authors:** Shruti Joshi, Seth Haney, Zhenyu Wang, Fernando Locatelli, Yu Cao, Brian Smith, Maxim Bazhenov

## Abstract

Distinguishing between nectar and non-nectar odors is challenging for animals due to shared compounds and changing ratios in complex mixtures. Changes in nectar production throughout the day and potentially many times within a forager’s lifetime add to the complexity. The honeybee olfactory system, containing less than 1000 principal neurons in the early olfactory relay, the antennal lobe (AL), must learn to associate diverse volatile blends with rewards. Previous studies identified plasticity between AL neurons but its role in odor learning remains poorly understood. We used a computational network model and live imaging of the honeybee’s AL to explore the neural mechanisms and functions of plasticity in the early olfactory system. Our findings revealed that when trained with a set of rewarded and unrewarded odors, the AL inhibitory network suppresses shared chemical compounds while enhancing responses to distinct compounds. This results in improved pattern separation and a more concise neural code. Our Calcium imaging data support these predictions. Analysis of a Graph Convolutional Network in machine learning performing an odor categorization task revealed a similar mechanism of contrast enhancement. Our model provides insights into how inhibitory plasticity in the early olfactory network reshapes coding for efficient learning of complex odors.

**Significance Statement:**

By combining computational modeling, machine learning, and analysis of calcium imaging data, we demonstrate that associative and non-associative plasticity in the honeybee antennal lobe (AL) - first relay of the insect olfactory system - work together to enhance the contrast between rewarded and unrewarded odors. Training the AL’s inhibitory network within specific odor environments enables the suppression of neural responses to common odor components, while amplifying responses to distinctive ones. This study sheds light on the olfactory system’s ability to adapt and efficiently learn new odor-reward associations across varying environments, and it proposes innovative, energy-efficient principles applicable to artificial intelligence.

## 1 Introduction

A honeybee can locate nectar-producing flowers by detecting floral aromas composed of many volatile compounds (Pichersky and Gershenzon, 2002; Negre et al., 2003). However, nectar-producing and non-producing flowers contain many of the same compounds, making it difficult for the honeybee to determine the map between odor sensing and reward prediction (Smith et al., 2006). This task is further complicated by the fact that nectar production may change within days and across locations, potentially many times within the foraging lifetime of a honey bee. Although the olfactory coding space is large (close to 1000 projection neurons in the honeybee antennal lobe, (Paoli and Galizia, 2021; Galizia, 2014)), mapping the sensory environment requires learning and relearning the association of reward with variable blends of volatile compounds. Nevertheless, honeybees can adapt to the variances in their environment owing to their keen ability to discriminate a wide range of olfactory stimuli (Menzel, 1990; Farooqui et al., 2003; Fernandez et al., 2009; Locatelli et al., 2013).

Different forms of plasticity are a ubiquitous feature of the early olfactory processing in the brains of both mammals and insects. In the neural networks of the mammalian Olfactory Bulb (OB) and insect Antennal Lobe (AL), both nonassociative (unsupervised) and associative (supervised) forms of plasticity have been described (Linster et al., 2009; Wilson and Linster, 2008; Fernandez et al., 2009; Locatelli et al., 2016). Together with the now well-established similarities in anatomical connectivity within the AL and OB networks (Hildebrand and Shepherd, 1997), it is clear that early olfactory processing in these phylogenetically very different groups of animals works in much the same way to augment olfactory processing. Both associative and nonassociative plasticity have been shown to affect the AL and have been implicated in changes in odor representations (Locatelli et al., 2013; Sudhakaran et al., 2012; Sinakevitch et al., 2017; Chen et al., 2015; Sachse et al., 2007; Das et al., 2011). The rich repertoire of foraging behavior and a relative simplicity of olfactory network makes the honeybee an excellent model for investigating early olfactory plasticity.

In this new study, we combined $Ca^{2+}$ imaging from honeybee AL with biophysically realistic computational modeling to characterize the role of inhibitory synaptic plasticity in the AL in improving odor discrimination after olfactory learning. We found that chemical compounds shared between rewarded and habituated odors are suppressed while distinct ones are enhanced after learning to increase contrast and reduce overlap between odor representations. This change in representation requires both associative and nonassociative plasticity on the inhibitory synapses between AL neurons. Analysis of the $Ca^{2+}$ imaging data revealed a change in odor representations after learning, supporting the model's prediction. Applying these ideas to the Artificial Neural Network (ANN) models trained to perform the odor categorization task revealed principles of contrast enhancement applicable to machine learning. In sum, we demonstrate the role of inhibitory synaptic plasticity in the AL for effective odor discrimination and propose a novel computationally efficient mechanism for performing categorization tasks.

## 2 Results

### Differential conditioning created distinct representations of odors *in vivo*

In our previous work (Locatelli et al., 2016) we used $Ca^{2+}$ imaging to test how representation of synthetic odor blends changes in the honeybee AL after differential conditioning (Figure 1A). Two synthetic classes of odors were based on varieties of the common snapdragon flower (*A. majus*): Potomac Pink (PP) and Pale Hybrid (PH) (Wright et al., 2005). Each class was a blend of volatile chemicals that recapitulated mean and variance of naturally occurring PH and PP flowers. These two classes contained the same volatile chemicals at different ratios that makes distinguishing them a difficult task.

We found that odors within each class created distinct representations in the AL via spatial activation of different sets of glomeruli (Figure 1B,C). Further, these representations became more distinct after differential training. Specifically, in absolute conditioning, honeybees were rewarded after responding to PH odors; in differential conditioning honeybees were also habituated to the PP odors (Locatelli et al., 2016) (Figure 1E). We found that the distance between representations of PH and PP odors increased after differential conditioning (Figure 1D). Although the change in Euclidean Distance is not significant, the correlation (similarity) between odor representations reduced significantly as shown in (Locatelli et al., 2016).

### Computational model of the mechanisms of differential conditioning

Here, we sought to investigate the mechanisms that produce differential learning between complex odors in the honeybee AL using a computational model. The model was a network of Hodgkin-Huxley type excitatory principal neurons (PNs) and inhibitory local neurons (LNs), representing honeybee AL network, and it was adapted from our previous work (Chen et al., 2015) (see Methods, Figure 2A). This model was previously developed to capture characteristic features of the AL dynamics, e.g., odor triggered LFP oscillations (Stopfer et al., 1997), and thus it enables us to study mechanisms of plasticity without compromising realistic AL dynamics which may play a role in shaping representations.

To model rewarded learning - appetitive conditioning - we simulated activity-dependent presynaptic facilitation at both the LN-PN and LN-LN synapses (see Methods) inspired by the finding that octopamine receptors activated by reward signal are localized on the inhibitory LNs in the AL (Sinakevitch et al., 2013). Octopamine is needed for appetitive olfactory learning (Hammer, 1993; Farooqui et al., 2003) and it has been shown to regulate inhibitory connections within the AL, affecting odor representations (Rein et al., 2013). The activation of octopamine receptors in LNs together with odor induced activity causes a rise in $Ca^{2+}$ that primes adenylyl cyclase to enhance cAMP and cAMP-dependent protein kinase activation that further regulates synaptic plasticity (Capogna et al., 1995; Han et al., 1998; Müller, 2002; Antonov et al., 2003). Therefore, we simulated the appetitive conditioning by increasing the synaptic weights of LN-PN and LN-LN synapses as the presynaptic LN was activated by the conditioned odor. Habituation was modeled as post-synaptic facilitation of LN-PN and LN-LN synapses, i.e., it depended on activity of postsynaptic LNs or PNs, based on the finding that habituation causes LN-PN facilitation in the fruit fly AL (Sudhakaran et al., 2012; Larkin et al., 2010).

To account for the fact that natural odors are composed of a blend of distinct volatile chemicals, we created inputs to the model combining activation of multiple distinct odor 'percepts'. A percept can be thought of as AL activation induced by specific chemical component of the odor (see (Locatelli et al., 2016) and Figure 5A below). Each precept in the model was represented by a distinct population of "virtual" ORNs making connections to simulated LNs and PNs. This effectively divided all the LNs and PNs into several percept groups (Figure 2B). A percept in the model could include more than one glomerulus consistent with an observation that a chemical can activate more than one glomerulus, each at a different

E

| Conditioning | Training | Testing/Imaging (10 trials) |
|---|---|---|
| Differential Conditioning | $PH_3$+ I $PP_2$- I $PP_4$- … $PP_3$- I $PH_3$+ I $PP_3$- | $PH_{1-6}$ I $PP_{1-6}$ |
| Absolute Conditioning | $PH_2$+ I MO- I $PH_4$+ … $PH_3$+ I MO- I $PH_2$+ | $PH_{1-6}$ I $PP_{1-6}$ |

**Fig. 1** Olfactory representations in the honeybee AL. A) Calcium imaging responses elicited by two different odors (PH1 and PP1) in a representative bee. Here, PH1 and PP1 belong to different classes of odors, with PH odors being presented along with a reward (rewarded odors) and PP odors presented without any reward (habituated odors). B) Position of the glomeruli within the Antennal Lobe, for which Calcium Imaging responses were measured. C) Mean activation across the different glomeruli elicited by the PH1 and PP1 odors. Glomeruli are ordered with the glomeruli most activated by PH1 at the top and the glomeruli least activated by PH1 at the bottom. The same ordering is used for PP1 odor as well to highlight the difference in the activation patterns between the two odors. D) Euclidean Distance of glomeruli activation between different odor classes (PH and PP) increases after differential conditioning compared to absolute conditioning. This indicates that the glomerular representation gets more separable after differential conditioning. E) Honeybee training protocol for differential and absolute conditioning. In differential conditioning, the bee was trained with rewarded (PH) and habituated (PP) odors presented in a randomized order. For absolute conditioning, the bee was trained with only rewarded odors. Mineral Oil (MO) was presented to the bee without any reward during absolute conditioning. Each trial lasts for 4s, with 500ms of odor presentation (Data from (Locatelli et al., 2016)). The same training protocol was used for training the computational model.

intensity, in vivo (Paoli and Galizia, 2021). As with real floral odors (Locatelli et al., 2016), model odors were composed of multiple overlapping percepts creating a difficult discrimination task.

### Differential training expands the coding space in the maximally discriminatory dimension

We trained our model using differential conditioning, where one class of odors $A = (A1, A2, ...)$ was rewarded and another $B = (B1, B2, ...)$ was habituated. Within each class, different odors were simulated by changing the width of Gaussian for individual percepts (Figure 2B). The model network was exposed to a sequence of $N = 30$ odors total presented in a randomised order, for a total of 60s (each odor presentation lasted 2s). The plasticity rule was selected based on which type of odor (i.e., rewarded or habituated) was presented. We found that the training led to significant changes in the PNs' population response for

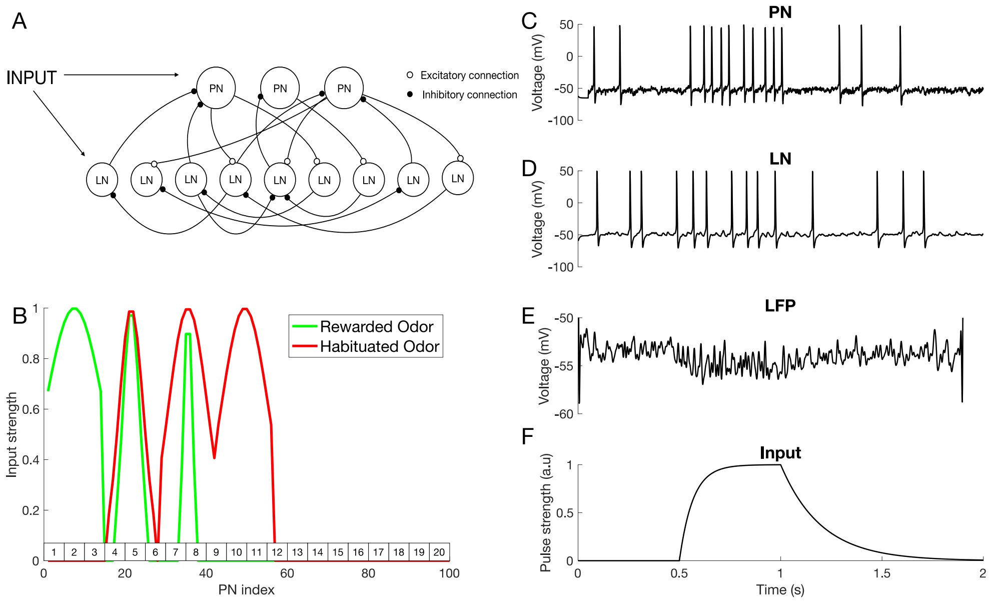

**Fig. 2** Computational model of the Antennal Lobe. A) Architecture of the AL network model, including excitatory PNs and inhibitory LNs (see Methods). B) Spatial pattern of odor stimuli for rewarded and habituated odors. The PNs are split into 7 groups, each corresponding to an odor percept. Percepts 1,2 and 3 are activated by the rewarded odor (green), while percepts 2,3 and 4 are activated by the habituated odor (red). The rectangles on top of the X-axis show the glomerulus number. C-D) Responses of a representative PN (C) and LN (D) to an odor input in the untrained network. E) Averaged PN activity (Local field potential). F) The 500ms time modulated current pulse which was provided as an inputs to individual neurons during odor stimulation (Gaussian noise was added on top of this input - see Methods).

the rewarded odors (Figure 3A). Specifically, neurons associated with percepts that were unique to the rewarded odors showed enhanced firing rates after training, whereas neurons associated with percepts in the overlap between rewarded and habituated odors were strongly suppressed. For habituated odors, plastic changes led to rather minor and non-specific reduction of the PN responses. These combined effects effectively stretched the AL coding space enhancing discrimination between rewarded and habituated odors.

We visualized effect of training using principal component analysis (PCA) (Figure 3B). We found that the trajectories of the naive response showed a relatively even distribution across PCA space. In contrast, after training, the sets of trajectories representing rewarded and habituated odors were strongly separated. We quantified this observation by measuring the time-averaged distance between odors. We found a significant increase in distance after differential training when comparing rewarded vs. habituated odors (p=1.9e-20) as well as when comparing odors within the same class, i.e., just rewarded or just habituated odors (p=1.88e-5) (Figure 3C). Both these results are in agreement with experimental data (Locatelli et al., 2016). The changes to odor representation in our model were due to changes in the inhibitory network, as these are the only synapses modified in the model. Analysis of the connectivity matrix after learning revealed a characteristic structure reflecting the structure of percepts in rewarded and habituated odors (Figure 3D).

Next, we individually tested the effect of associative vs non-associative plasticity on the ability of the trained network to distinguish between the rewarded and habituated odors by selectively applying only pre or post-synaptic plasticity and measuring the distance between the learned odor representations. In Figure 4A, we show the Euclidean Distances between rewarded and habituated representations for different conditions - (i) Naive (pre-synaptic and post-synaptic plasticity are both off), (ii) Associative only (pre-synaptic plasticity is on, post-synaptic plasticity is off; absolute conditioning), (iii) Non-Associative only (pre-synaptic plasticity is off, post-synaptic plasticity is on), and (iv) Both (pre-synaptic and post-synaptic plasticity are both on; differential conditioning). We found that the largest increase in Euclidean distance is seen when both associative and non-associative plasticity are active (p=1.9e-20), followed by Non-associative only (p=1.5e-19) and Associative only (p=5.45e-18) in that order (Figure 4A, left)

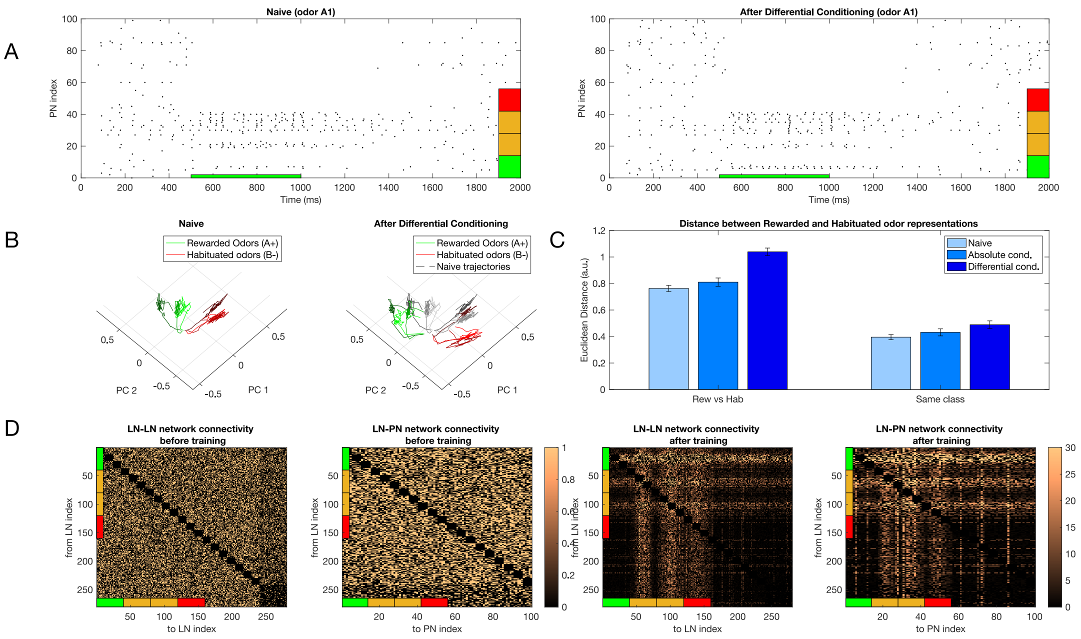

**Fig. 3** Change in representation of simple odors after learning. A) Raster plot of the PN population response to rewarded odor (odor $A1$) before (left) and after (right) differential conditioning. Note, that activity of PNs activated by both rewarded and habituated odors (denoted by orange percepts) decreased while the activity of PNs activated uniquely by the rewarded odor (denoted by the green percept) increased. Red indicates percepts uniquely activated by the habituated odor. B) Dynamical trajectory of the spatiotemporal odor responses in two-dimensional (2D) PCA space. The green lines show trajectories for 3 Rewarded Odors and the red ones for 3 Habituated Odors. After training, the trajectories of the rewarded and habituated odors shift away from each other. Black lines on the right plot indicate naive trajectories. C) Left, the Euclidean Distance between the rewarded and habituated odors for naive network (light blue), and after absolute (middle tone blue) and differential (dark blue) conditioning. Right, the distance between odors from the same class. The Euclidean Distance is calculated for each pair of odors and then averaged over all the pairs of odors. The error bars show the standard deviation of the Euclidean Distance over trials. D) Connectivity of the inhibitory networks (LN-LN and LN-PN) before (left) and after (right) training. Connectivity after differential conditioning shows a grid-like structure which encodes the relationship between percepts belonging to the rewarded and habituated odors. The colorbars show the final value of the weight divided by its initial value.

when compared with the naive case. We found small but significant increases in the Euclidean Distance measured between odors of the same class for all 3 conditions compared to naive (Figure 4A, right). The structure of LN-LN and LN-PN networks after learning for the Associative only and Non-Associative only cases is shown in Figure 4B and C, respectively. As expected, it reveals changes in only subset of connections (compare to Figure 3D). In Figure 4C right plot, we can see some bright vertical lines showing increased inhibition to certain PNs which are not activated directly by the odor (vertical lines between PN 60 and 80 and 80 and 100). These lines appear because some PNs have a high background firing which seems to increase even more after Non-associative (only) training.

### Training with realistic odorants also produces contrast enhancement

To ensure that the effects we have shown so far are not due to the specific construction of the model odors, we created a new set of odors where fractions of percepts were aligned with the fractions of the measured volatile chemicals present in each of the odor blend categories (i.e., PH1, PH2,.. and PP1, PP2,...) that were used in behavioral experiments in (Locatelli et al., 2016). These PH and PP odor classes were created by calculating the proportion of the chemical components present in the blends and setting the activation of each percept according to it (Figure 5A, see Methods for details). Training these odors using differential conditioning (PH was rewarded and PP was habituated) also produced an expansion of the coding space (Figure 5C); both the distance between odors from the different classes (i.e., rewarded and habituated, p=4.53e-21) and odors from the same class (e.g., PH1 vs PH2,..., PP1 vs PP2,..., p=3.16e-15) increased (Figure 5B), in line with our previous results obtained with simpler model (Figure 3B). This finding has been also seen in vivo (Locatelli et al., 2016). There, decrease in similarity of representations was found

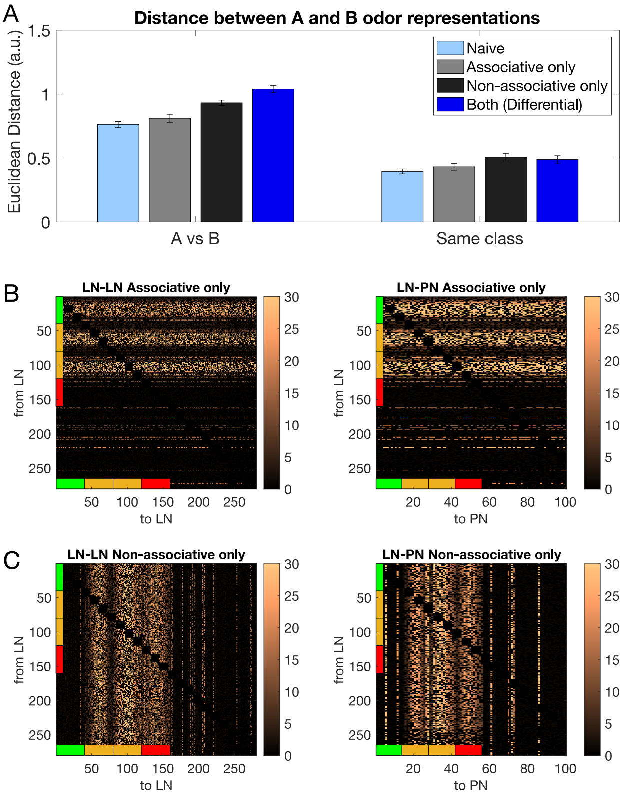

**Fig. 4** Connectivity of the inhibitory network after associative only and non-associative only training. A) Left shows the Euclidean Distance between rewarded and habituated odors increases after associative (gray) as well as non-associative (black) training compared to naive (light blue). The highest increase is found when training included both associative and non-associative plasticity (dark blue) - differential conditioning. Right shows Euclidean Distance calculated for odors belonging to the same class. B) The LN-LN and LN-PN network after associative only training. C) The LN-LN and LN-PN network after non-associative only training. The colorbars show the value of the weights after training divided by the initial weights.

between different PH and PP odors, indicating that differential conditioning might make recognition of individual rewarded odors easier.

Inspired by our modeling results, where we found that representations of PNs activated by percepts that are unique to the trained odors are enhanced and representations of PNs activated by percepts that are common to many odors are suppressed leading together to increase in Euclidean Distance between odors (Figure 5B), we sought to quantify this effect in vivo. Using the $Ca^{2+}$ imaging data from honeybee (collected as described in (Locatelli et al., 2016)), we created a uniqueness score for each glomerulus. This was done by calculating for each glomerulus the difference between its activation by the rewarded odor and its activation by the habituated one (see Methods) Here, we consider the comparison between odor representations obtained after differential conditioning vs absolute conditioning, since we do not have experimental recordings for the naive case. We found a strong correlation between the uniqueness score of a glomerulus and the change in its activity due to differential conditioning compared to absolute conditioning (Figure 5D). Specifically, glomeruli tended to increase their activity after training if they were unique in the representation of the rewarded odor and vice versa, in agreement with what our model predicts. This effect of expanding the coding space, that aligns with the uniqueness of the percepts, we term *contrast enhancement*.

### The inhibitory AL network adapts to discriminate between odors in a new environment

As honeybees move from one environment to another, the odors associated with reward and habituation may change. The animal needs to learn the association of these new odors with the reward contexts

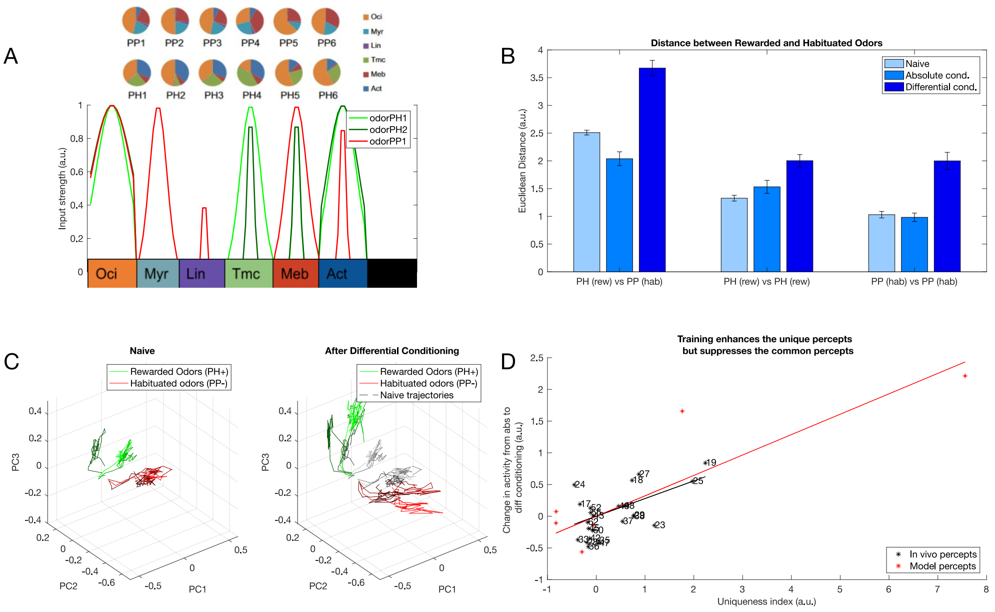

**Fig. 5** Contrast enhancement with complex odors based on in vivo data. A) Odors obtained from in vivo experiments (PH,PP) converted into inputs to the model (see Methods for details). B) Euclidean Distance between rewarded (PH) and habituated (PP) odors increases after differential conditioning (dark blue) compared to the naive network (light blue) and absolute conditioned network (middle tone blue). Distance between odors belonging to same rewarded class (e.g., PH1 vs PH2, PH1 vs PH3, ...) increases slightly after absolute conditioning (middle tone blue) and significantly after differential conditioning (dark blue). Distance between the odors belonging to the habituated class does not increase after absolute conditioning (middle tone blue) but increases significantly after differential conditioning (dark blue). These results match experimental data. C) The dynamical odor trajectory in the 3D PCA space shows the 3 rewarded (PH1, PH2, PH3) and 3 habituated odor (PP1, PP2, PP3) representations shifting in the opposite direction which makes odors more discriminable. D) Contrast enhancement based on $Ca^{2+}$ imaging data (black) and model (red). After differential conditioning, activity in the glomeruli/percepts which have a high uniqueness index (i.e., uniquely activated by the rewarded odor) increases while activity in the glomeruli/percepts which have a low uniqueness index (i.e., activated by both rewarded and habituated odors) decreases ($R^2$=0.3751, p=0.0015 for in vivo percepts and $R^2$=0.7651, p=0.0225 for model percepts). Note, similar trend in vivo and in the model.

present in the new environment. Changes in nectar production throughout the day and potentially many times within a forager's lifetime add to the complexity. The inhibitory network, therefore, must be flexible enough to learn representations for the new odors and successfully discriminate between them without immediately forgetting the old environment.

The odors from the new environment (we will call it Env2) may or may not overlap with the odors from the first environment (Env1) the animal has learned already. Below, we discuss four cases based on the overlap between the odors of the two environments. As before, each environment has two classes of odors (e.g., class P - rewarded, and class Q - habituated), each class containing 10 odors.

Case 1: The odor classes from Env1 (P and Q) do not overlap with the odor classes from Env2 (S and T). Case 2: Both rewarded and habituated odor classes from Env1 and Env2 overlap. Here, Env2 consists of odor class M (overlaps with class P) and odor class N (overlaps with class Q). Case 3: One odor class from Env2 (class M) overlaps with the rewarded odor class from Env1 (class P), and the second odor class from Env2 (class S) does not overlap with any odors from Env1. Case 4: One odor class from Env2 (class N) overlaps with the habituated odor class from Env1 (class Q), and the second odor class from Env2 (Class S) does not overlap with any odor from Env1.

The structure of each of the odor classes used in experiments here is shown in Figure 6, on top of the plots. Each class was based on three percepts and, as before, individual odors within each class were obtained by varying activation of individual percepts. The reward structure for the first environment is always odor class P is rewarded and class Q is habituated (P+Q-). Each case is further divided into two sub-cases based on which odor class from the second environment is rewarded and which is habituated.

Case 1 (Figure 6A): When there is no overlap between odors from Env1 and Env2, the Euclidean distance between the representations of classes P and Q increases after training on Env1 (P+Q-) and remains high after training on Env2. In contrast, the distance between odors S and T only increases after training on Env2, regardless of the reward structure (S+T- or S-T+) (Figure 6A). This finding suggests that the network can effectively learn a new environment and distinguish between odors from different environments when there is no interference.

Cases 2 and 3 (Figure 6B,C): When rewarded odors from Env1 overlap with rewarded odors from Env2, the Euclidean distance between odor classes P and Q from Env1 further increases after training on Env2 (Figure 6B and C, left plots). This increase occurs because training on Env2 reinforces the associations learned in Env1, as the overlapping percepts from odors in both environments have the same reward associations. However, if the reward structure of Env2 is reversed, the distance between odor classes P and Q decreases after training on Env2 (Figure 6B and C, right plots), indicating that the network must unlearn the associations from Env1 to learn the new associations for Env2.

Interestingly, when both rewarded and habituated odors from two environments overlap but have opposite reward associations (Case 2), a single training session on Env2 is insufficient to increase the distance between Env2 odors (Figure 6B, right plot). In contrast, if only the habituated odors from Env2 (class M) overlap with the rewarded odors from Env1 (class P) (Case 3), the distance between the two odor classes increases after training on Env2, suggesting a partial reversal in reward associations (Figure 6C, right plot).

Case 4 (Figure 6D): When odors from Env2 (class N) only overlap with the habituated odors from Env1 (class Q), the distance between representations of odors P and Q decreases slightly after training on Env2 (Figure 6D, first set of bars). This decrease in distance is more pronounced when odor N is being rewarded instead of habituated (Figure 6D, right plot, first set of bars), demonstrating that the network must partially unlearn the associations in the overlapping percepts between odors N and Q to adapt to Env2. For both reward structures of Env2 (S+N- and S-N+), the distance between representations of odors S and N does not change after training on Env1 but increases after training on Env2 (Figure 6D, second set of bars).

The findings of this experiment provide insights into how an inhibitory AL network adapts to different reward structures and odor overlaps across multiple environments. The network can effectively distinguish between odors from different environments when there is no overlap. However, when odors overlap, particularly when the reward structures are reversed, the network must unlearn and relearn associations. The extent of unlearning and relearning depends on the specific overlap scenario and the reward associations of the overlapping odors.

Notably, the overlap between an odor from Env2 and the rewarded odor from Env1 has a more significant impact on the representations and distances compared to an overlap with the habituated odor from Env1. This observation suggests that the network is more sensitive to changes in the reward associations of previously rewarded odors than to changes in the associations of previously habituated odors.

Furthermore, the results show that it takes longer for the network to learn the second environment when the reward structures are reversed, which is consistent with experimental studies on reversal learning (Pavlov, 1928; Komischke et al., 2002). Latent inhibition refers to the observation that prior exposure to a stimulus without any associated reward can slow down the subsequent learning of associations involving that stimulus (Chandra et al., 2010; Bazhenov et al., 2013). In the context of this experiment, the prior learning of reward associations in Env1 can interfere with the learning of new associations in Env2 when the odors overlap but have opposite reward structures, resulting in slower learning and adaptation to the new environment.

### Graph convolution neural networks and inhibitory learning converge on similar solutions

Can the principles learned from olfaction be exploited to improve performance of artificial neural networks (ANNs)? The encoding of odors in the AL takes place by learning the relationships between the components of the odors and the reward associated with the odors. Graph convolutional network (GCN) is a class of ANNs that take graph data as the input and aggregates information from neighboring nodes of the graph to perform a task (e.g., classification) (Duvenaud et al., 2015; Kipf and Welling, 2016). This enables the GCN to utilize the relationships between nodes to improve the classification performance (Kipf and Welling, 2016). Hence, GCNs can be considered analogous to the AL, in that they encode relationships between different components of the input and utilize these relationships to categorize the inputs. To enable classification, GCN network was followed by simple fully connected network, that can be seen as analogous to subsequent processing centers of the olfactory system, such as mushroom bodies.

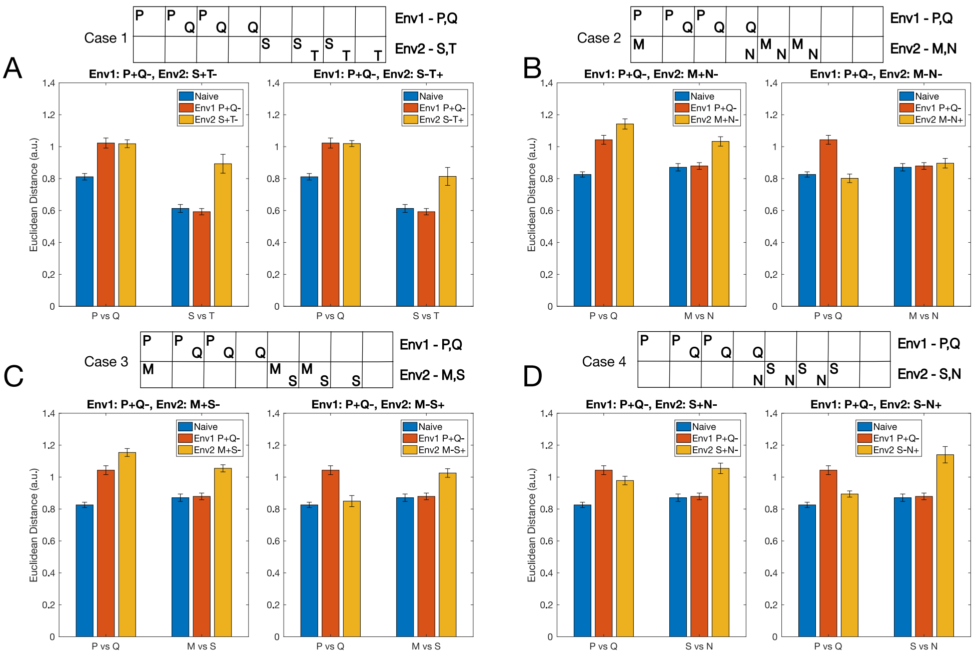

**Fig. 6** Learning by the AL inhibitory network in different environments. The network is initially trained on environment 1 (Env1) containing rewarded odor class P and habituated odor class Q. Subsequently, the network is trained on a new environment 2 (Env2), containing new odor classes (e.g., S and T). Bars show the distances between odor classes P and Q (left set of bars in each subplots) and between odor classes from a new Env2 (right set of bars) at different stages: naive (blue), after training on Env1 (orange), and after subsequent training on Env2 (yellow). Each case (1-4) represents a different scenario of odor overlap between Env1 and Env2, with sub-cases (left and right subplots for each case) depicting different reward structures in Env2 (e.g., S+T- vs S-T+). A) Case 1: No odor overlap between Env1 and Env2. B) Case 2: Both odors from Env2 overlap with odors from Env1. C) Case 3: One odor from Env2 overlaps with the rewarded odor from Env1. D) Case 4: One odor from Env2 overlaps with the habituated odor from Env1. The boxes at the top illustrate the distinct percepts triggered by the odors in each environment.

Here, we compared results obtained using our biophysical model of the honeybee AL network to a GCN. In order to do that, we trained each network on chemical gas sensor data (Vergara et al., 2012), where the biophysical network model was trained via the inhibitory plasticity (as described before) (Figure 7) and the GCN was trained using backpropagation (see Methods) (Figure 8). The numeric features in this dataset, which included response magnitude, On and Off time constants, and others (see Methods), were used directly to train the GCN model. In the biophysical model, we used three features (response magnitude and On and Off time constants) from the data to construct input pulses to model odor stimulation (Figure 7A).

We selected half of the odors to be associated with the reward and the other half to be habituated. Learning in the biophysical model increased the discriminability between the rewarded and habituated odors as indicated by the increase in Euclidean Distance (Figure 7B).

The GCN network (Figure 8A) was able to identify which class an odor belonged to (i.e., rewarded or habituated) with 88% accuracy. Importantly, we found that both the GCN and the biophysical model showed the same characteristics in the changes to representations of odors. Specifically, units that represent components that are unique to an odor were enhanced and units that represent components common to many odors were suppressed (Figure 8B). Both the top-down approach, rooted in backpropagation, and the bottom-up approach, grounded in local biological learning rules, converge to the same contrast enhancement strategy. This result highlights the effectiveness of contrast enhancement as a computational strategy for accomplishing categorization tasks.

## 3 Discussion

In vivo recordings from the honeybee's antennal lobe (AL) have found that separation among patterns of the neural activity representing odor blends from different varieties of flowers improves after differential reinforcement for each variety (Locatelli et al., 2016). However, the mechanisms behind this phenomenon

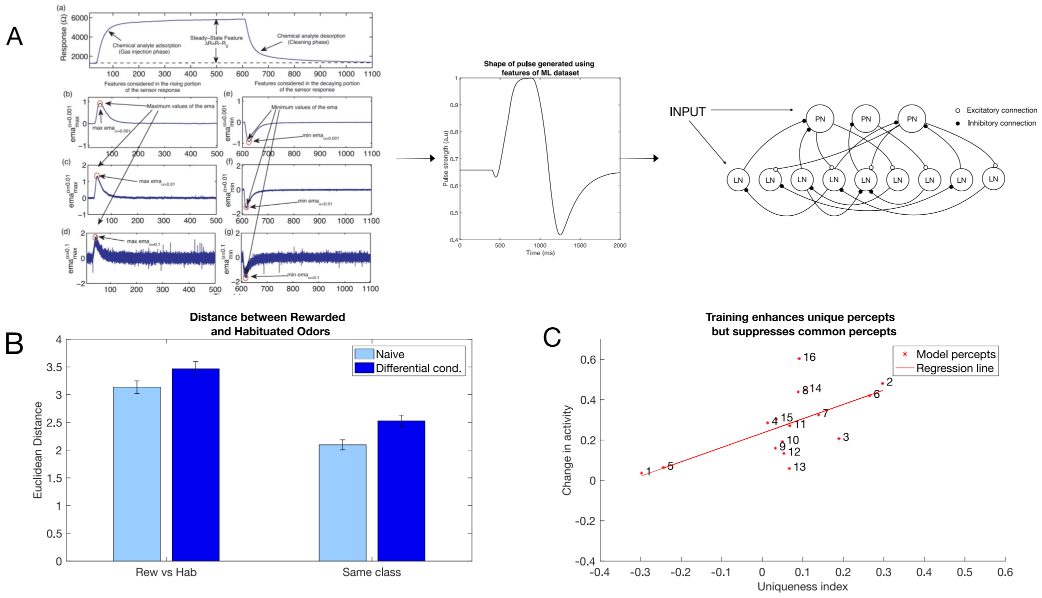

**Fig. 7** Contrast enhancement in a large network model with inputs generated from chemical gas sensor data (Gas Sensor Array Drift Dataset from (Vergara et al., 2012)). A) The features extracted from the response curves are converted into a pulse that is then fed into the larger biophysical model as an input. B) Euclidean distance between and within classes increases after differential conditioning training. C) Contrast enhancement analysis for the large biophysical network using chemical gas sensor data revealed similar results to the smaller biophysical model and in vivo data ($R^2 = 0.416$, p=0.007)(compare to Fig. 5D).

remain poorly understood. The primary goal of this study was to uncover the plasticity mechanisms that lead to the modification of neural representations of natural odor blends in the early olfactory system - honeybee AL - using a combination of computational modeling, machine learning and $Ca^{2+}$ imaging.

Natural odors are generally blends of several chemical components (Pichersky and Gershenzon, 2002). These odors are present in different concentrations, and nectar production may change from season to season and environment to environment. Although the olfactory coding space is large, optimally mapping the sensory environment requires learning and relearning the association of reward with variable blends of volatile compounds (Pichersky and Gershenzon, 2002). We found that, through a combination of associative and non-associative plasticity in the inhibitory AL network, the common components in the neuronal representations of the rewarded and habituated odors decrease their activity, while the components unique to rewarded odors increase their activity. This leads to a decrease in overlap between the representations of the odor classes, while also making the representation of the rewarded odors more compact. This ”contrast enhancement” effect was predicted by our biophysical model and model predictions were confirmed by the $Ca^{2+}$ imaging data from the honeybee AL.

We also found that when subsequently stimulated by odors from a different environment, the inhibitory AL network learned the odor-reward associations from the new environment while preserving associations from the old environment. To test the application of these principles to Artificial Intelligence, we developed and trained a machine learning model based on the Graph Convolutional Network (GCN) that performed the same task of categorizing complex odors. Analyzing the activity of the units in the GCN, we found a similar strategy of changes in the representation of the rewarded odors as observed in the biophysical model and in vivo data.

Previous works have successfully applied computational network models to study mechanisms of odor-induced synchronization (Bazhenov et al., 2001b; Sanda et al., 2016; Assisi et al., 2011; Ito et al., 2009; Bazhenov et al., 2001a) and neuronal plasticity (Locatelli et al., 2013; Linster and Smith, 1997; Chen et al., 2015; Bazhenov et al., 2013; Finelli et al., 2008; Bazhenov et al., 2005; Assisi et al., 2020) in the early olfactory system of insects. The study by (Locatelli et al., 2013) used both firing rate and conductance-based models to explore non-associative plasticity in the AL. The model in (Chen et al., 2015) revealed that stimulus-specific changes in synaptic inhibition are sufficient to explain shifts in odor representations after olfactory learning.

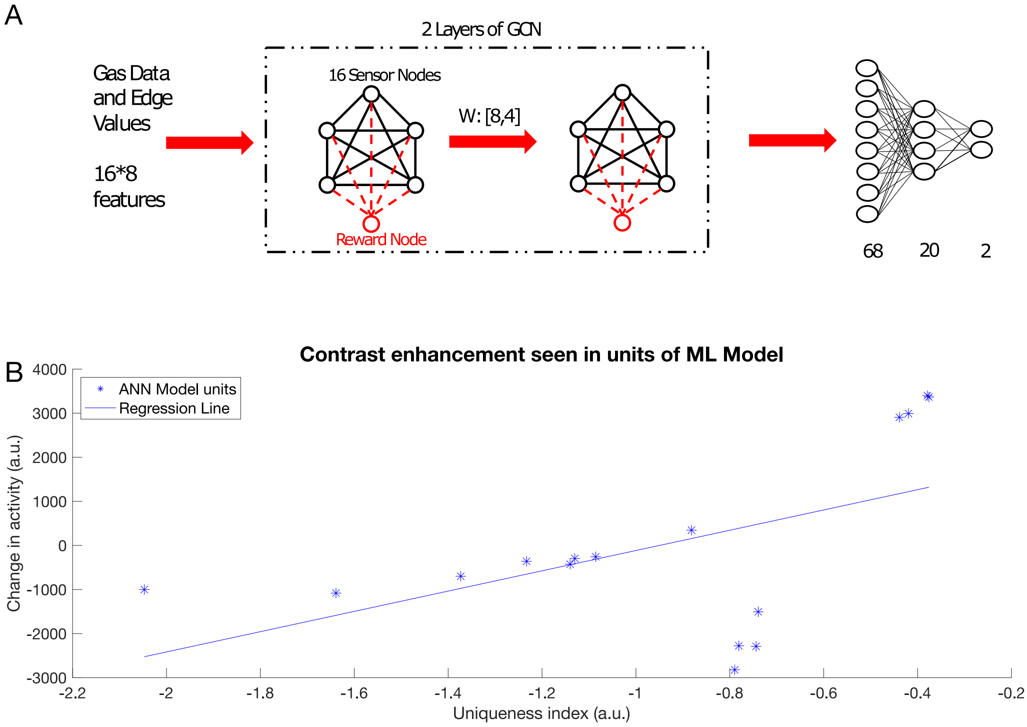

**Fig. 8** Contrast enhancement in a machine learning model. A) Schematic of the GCN model. The model receives features from the Gas Sensor Array Drift Dataset and processes it through two graph convolutional layers. The graph is then unrolled into a vector and sent through two fully connected layers for categorization. Training weights between GCN layers can be thought of as analogous to the biophysical model training. B) Change in activity in the graph nodes in the first and second layer of the GCN vs uniqueness index of that node ($R^2 = 0.276$, p=0.031). Note contrast enhancement: nodes with higher uniqueness tend to increase the activity and those with lower uniqueness index tend to decrease the activity.

Our new study revealed that the AL network might utilize inhibitory synaptic plasticity to optimize its responses to odors associated with rewards in a given odor environment. Considering the limited neuronal resources of the insect olfactory system and the complexity of the tasks it needs to solve, our study proposes a novel strategy by which the early olfactory system can facilitate odor processing at downstream levels, focusing on odors relevant for survival. The model predictions are in agreement with imaging studies in the honeybee (Faber et al., 1999; Locatelli et al., 2016; Sachse and Galizia, 2002; Sandoz, 2003) and other insects (Wilson et al., 2004) showing that odor representations in the AL change after differential conditioning, with rewarded and unrewarded odor representations becoming less correlated. These findings support the notion that the increase in the separation of odor representations in the AL in vivo occurred through contrast enhancement, thus validating the prediction made by our model. The main principles of nonassociative and associative plasticity operating in the first relay for olfactory coding are similar in the brains of insects and vertebrates (Linster et al., 2009; Wilson and Linster, 2008; Fernandez et al., 2009; Locatelli et al., 2016, 2013) and our model predictions can be generalized beyond insect olfaction.

Honeybees have a rich repertoire of learning behaviors to odors that range from nonassociative through associative and operant conditioning (Hammer and Menzel, 1995; Smith et al., 2006). Biogenic amines have been shown to play an important role in reinforcement during olfactory learning (Hammer, 1993; Hammer and Menzel, 1998; Aso et al., 2010). In the honeybee, a cluster of cells at the base of the brain called Ventral Unpaired Medial (VUM) cells receive input from sucrose (US)-sensitive taste receptors (Hammer, 1993; Laurent et al., 1998; Bicker and Kreissl, 1994; Abel et al., 2001; Sinakevitch et al., 2005).

The outputs from VUM cells spread broadly throughout association areas of the brain, including the AL and the MB, where they release the biogenic amines octopamine and tyramine as the reinforcement signal. Immunological studies have shown that the octopamine receptor (AmOA1) is expressed alongside GABA receptors in the honeybee AL (Sinakevitch et al., 2013). Other studies (Rein et al., 2013) have shown that octopamine modulates odor representations in PNs by regulating inhibitory connections in the AL. Repeated or prolonged exposure to a stimulus without any reinforcement reduces the behavioral response to those odors, a process known as habituation (Thompson and Spencer, 1966; Bazhenov et al., 2013; Chandra et al., 2010), which leads to latent inhibition (Chandra et al., 2010; Latshaw et al., 2023). Studies in the fruit fly AL have revealed that habituation arises from the facilitation of inhibitory LN to PN synapses involved in odor representation (Larkin et al., 2010; Sudhakaran et al., 2012).

The model predicts that both associative and non-associative plasticity are needed to explain in vivo data. The suppression of the common component in the rewarded odors was driven by an increase in the inhibitory LN to PN connection strength, caused by postsynaptic (non-associative) plasticity. This effect resulted from the firing of PNs triggered by the habituated odor, aligning with the findings of (Locatelli et al., 2013) and (Chen et al., 2015). These studies demonstrated that non-associative inhibitory plasticity from LNs to PNs played a crucial role in 'filtering out' components of the habituated odor after honeybee was exposed to the odor without receiving a reward. The increase in the activity of the unique component in the rewarded odors occurred due to both presynaptic and postsynaptic facilitation, leading to increase in inhibition of the LNs that were inhibiting PNs representing the unique component (i.e., PNs' disinhibition). A similar effect, an increase in the activity of the glomeruli for the rewarded but not the habituated odor, was also observed in (Faber et al., 1999). Together, these two mechanisms contributed to shifting the PN responses for the rewarded odor away from the habituated odor.

The Mushroom Body (MB), the next layer in olfactory processing in insects, is considered a major center for associative learning and olfactory memory (Hammer and Menzel, 1998). Information from the PNs in the AL is relayed to the MB, where Kenyon Cells (KCs) represent odors with a sparse firing patterns (Stopfer et al., 2003; Perez-Orive et al., 2002). Remarkably, approximately 800 PNs synapse onto around 170,000 KCs (Mobbs and Young, 1997; Gronenberg, 1986), significantly increasing the dimensionality of the odor representation space. Experimental studies have shown that KC responses are influenced by associative reinforcement learning, which stabilizes odor representations in the MB. In contrast, odor presentations without any reinforcement weaken their representation in the MB (Szyszka et al., 2008). Modeling studies (Rajagopalan and Assisi, 2020) have also found that the categorization of odors in the MB depends on changes in PN responses caused by conditioning. Our study suggests a potential mechanism for this change in KC responses. The rewarded odor triggers more population-specific AL activity after training, therefore, the KCs receiving input from these PNs display more reliable firing, as suggested by (Szyszka et al., 2008).

Artificial Intelligence has made rapid progress in recent years in solving tasks such as image classification, speech recognition, natural language processing. Although it has now grown into a separate field, AI can trace its roots back to neuroscience with the earliest artificial neural networks trying to mimic information processing in the brain (Hassabis et al., 2017). Currently, efforts are being made to incorporate biological principles to develop new generation of AI (Hayes et al., 2021; Kudithipudi et al., 2022). Graph Neural Networks (GNNs) have been developed to work with graph data. There have been significant advances in GNNs, increasing their capabilities and expressive power (Kipf and Welling, 2016; Reiser et al., 2022). In one study GNNs have been used to learn a generalizable perceptual representation of odors (Sanchez-Lengeling et al., 2019). Here, we developed a Graph Convolutional Network (GCN) model as an artificial parallel to the insect Antennal Lobe. We trained this network to perform an odor categorization task and analyzed the activity of the artificial neurons in the first and second layers of the GCN. This analysis revealed that the contrast between representations of different odor classes increased based on the same mechanism described for the biophysical model, i.e., units that represent components that are unique to an odor were enhanced and units that represent components common to many odors were suppressed. Together, these results suggest that contrast enhancement is an efficient strategy for performing a categorization task.

In summary, our study has unveiled novel circuit-level mechanisms of olfactory learning in the honeybee AL, which enhance odor discrimination. This learning paradigm relies on inhibitory network plasticity, enabling the reshaping of the coding space to align with the current task and environment. These findings suggest an effective computational strategy for the perceptual learning of complex natural odors, achieved through the modification of the inhibitory network during early sensory processing.

## 4 Methods and Materials

### Animals and calcium imaging experiments

Pollen forager honey bees (Apis mellifera) were collected from regular hives and restrained in individual harnesses suited for olfactory conditioning and calcium imaging recordings. Appetitive olfactory conditioning followed standard protocols (Smith and Burden, 2014). Two groups of bees received different conditioning protocols using odor blends that mimicked the natural odor variation within and between two varieties of Snapdragon flowers, Pale Pink (PP) and Pale Hybrid (PH) (Wright et al., 2005; Locatelli et al., 2016). One group of bees was subject to differential conditioning (PH+/PP-) by rewarding with 1M sucrose solution odor blends from the PH variety and not rewarding blends of the PP variety. The second group of bees (PH+/MO-) was trained rewarding the same PH blends as for the first group, but the odor solvent mineral oil (MO) was used instead PP blends for unrewarded trials. Thus, the difference between groups was in the unrewarded trials. Eight hours after conditioning, uniglomerular projections neurons of the antennal lobe were stained by backfilling with the calcium sensor dye fura-dextran (potassium salt, 10.000 MW, ThermoFisher Scientific) (Sachse and Galizia, 2002). Calcium imaging was performed on the next day. The head-capsule was opened and rinsed with Ringer solution (130 mM NaCl, 6 mM KCl, 4 mM MgCl2, 5mM CaCl2, 160mM sucrose, 25mM glucose, 10mM HEPES, pH 6.7, 500 mOsmol; all from Sigma-Aldrich). Glands and trachea covering the antennal lobes were removed. Only one antennal lobe per bee was used for calcium imaging. Calcium imaging was done using a CCD camera (SensiCamQE, T.I.L.L. Photonics) mounted on an upright fluorescence microscope (Olympus BX-50WI, Japan) equipped with a 20x objective, NA 0.95 (Olympus), 505 DRLPXR dichroic mirror, and 515 nm LP filter (TILL Photonics). Monochromatic excitation light provided by a PolichromeV (TILL Photonics) alternated between 340 and 380 nm. Images were recorded at a sampling rate of 8 Hz. Spatial resolution was 172 x 130 pixels, after a binning of 8 x 8 on a chip of 1376 x 1040 pixels, resulting in a spatial sampling of 2.6 µm per pixel side. Each data set consisted in a double sequence of 80 images, obtained at 340 and 380 nm excitation light respectively (Fi340, Fi380, where i is the number of images from 1 to 80). Calcium signals were calculated using a ratiometric method: for each pair of images Fi we calculated Ri = (Fi340/Fi380) x 100 and subtracted the background Rb that was obtained by averaging the Ri values 1 sec before odor onset. The analysis was based on the calcium signals from the same 24 glomeruli identified in all bees (figure 1C) (Galizia et al., 1999). The glomerular activation was considered as representing the activity of uniglomerular PNs, as only these neurons in the AL were stained. The odor delivery device consisted of a main charcoal filtered air stream (500 ml/min) and fifteen identical odor channels, each composed of one valve and one odor cartridge. The odor cartridges consisted of a 1 ml syringe containing a paper strip loaded with 10 ul odor solution. A three-way valve (LFAA1200118H; The LEE Company) controlled the airflow through the odor cartridge. Opening of the valves was synchronized with the optical recordings by the acquisition software TILLVisION (TILL Photonics). During stimulation with odor, 4 ml of air loaded with the odor was added to the main air stream during 4 seconds. Calcium imaging analysis was designed to determine how both trainings protocols affect the neural representation of PH and PP blends. The calcium activity measured between 375 and 625 ms after odor onset was averaged, thus the activation pattern elicited by each odor was reduced to a single vector of 24 elements (glomeruli) (see figure 1A for examples). This time interval includes the time points in which odor representations reach the maximal distance after odor onset. The similarity between 2 glomerular odor patterns was calculated as the Euclidean Distance between the respective 24-dimensional vectors. As we measured glomerular activity patterns elicited by 6 PH blends and 6 PP blends, we could calculate for each animal the average distance between 36 pairs of PH-PP blends. This was done for 10 absolute conditioned bees and 7 differentially conditioned bees. This data has been previously published in (Locatelli et al., 2016).

### Computational Modeling

We constructed a biophysical model of the honeybee Antennal Lobe (AL), containing 100 excitatory Projection Neurons (PNs) and 280 inhibitory Local Interneurons (LNs). Each PN and LN was modeled by a single compartment that included voltage- and calcium-dependent currents described by Hodgkin-Huxley kinetics (Hodgkin and Huxley, 1952). The model here was adapted from our previous studies (Haney et al., 2018), (Chen et al., 2015) and is available for download from the Bazhenov lab website. Model equations were solved using a fourth order Runge-Kutta method with an integration time step of 0.04ms.

### Membrane Potentials

PN and LN membrane potential equations (Hodgkin and Huxley, 1952) are given by

$$C_m \frac{dV_{PN}}{dt} = -g_L(V_{PN}-E_L)-I_{Na}-I_K-I_A-I_T-I_h-g_{KL}(V_{PN}-E_{KL})-I_{GABA_A}-I_{\text{slow-inh}}-I_{nACh}-I_{stim}$$

$$C_m \frac{dV_{LN}}{dt} = -g_L(V_{LN}-E_L)-I_{Na}-I_K-I_T-g_{KL}(V_{LN}-E_{KL})-I_{GABA_A}-I_{nACh}-I_{stim}$$

The passive parameters are as follows: For PNs, $C_m = 2.9 * 10^{-4}\mu F$, $g_L = 0.01mS/cm^2$, $g_{KL} = 0.012mS/cm^2, E_L = -70mV, E_{KL} = -95mV$.

For LNs, $C_m = 1.43 * 10^{-4}\mu F$, $g_L = 0.05mS/cm^2$, $g_{KL} = 0.018mS/cm^2, E_L = -70mV, E_{KL} = -95mV$. An external DC input was introduced to each neuron through $I_{stim}$.

The intrinsic currents, including a fast sodium current, $I_{Na}$, a fast potassium current, $I_K$, a transient potassium A-current, $I_A$, a low threshold transient $Ca^{2+}$ current, $I_T$, a hyperpolarization activated cation current $I_h$ are given by equation

$$I = gm^M h^N(V-E)$$

where the maximal conductances for PNs are $g_{Na} = 90mS/cm^2, g_K = 10mS/cm2, g_A = 10mS/cm^2, g_T = 2mS/cm^2, g_h = 0.02mS/cm^2$. The maximal conductances for LNs are $g_{Na} = 100mS/cm2, g_K = 10mS/cm^2$, and $g_T = 1.75mS/cm^2$.

For all cells, $E_{Na} = 50mV, E_K = 95mV$, and $E_{Ca} = 140mV$. The gating variables $0 \le m(t), h(t) \le 1$ satisfy the following:

$$\frac{dm}{dt} = \frac{m_\infty(V)-m}{\tau_m(V)}$$
$$\frac{dh}{dt} = \frac{h_\infty(V)-h}{\tau_h(V)}$$

where the steady state (e.g., $m_\infty(V)$) and relaxation times (e.g., $\tau_h(V)$) are derived from experimental recordings of the specific ionic currents. These distinct voltage-dependent functions are given in (Chen et al., 2015). For all cells, intracellular $Ca^{2+}$ dynamics were described by a simple first-order model as follows:

$$\frac{d[Ca^{2+}]}{dt} = -A \cdot I_T - \frac{[Ca^{2+}]-[Ca^{2+}]_\infty}{\tau}$$

where $[Ca^{2+}]_\infty = 2.4 \cdot 10^{-4}mM$ is the equilibrium of intracellular $Ca^{2+}$ concentration, $A = 5.2 \cdot 10^{-5}mM \cdot cm^2/(ms \cdot \mu A)$ and $\tau = 5ms$.

### Synaptic Currents

Fast GABA and cholinergic synaptic currents to LNs and PNs were modeled by first-order activation schemes (Destexhe et al., 1994). Fast GABA and cholinergic synaptic currents are given by:

$$I_{syn} = g_{syn}[O](V-E_{syn})$$

where the reversal potential is $E_{nACh} = 0mV$ for cholinergic receptors and $E_{GABA} = -70mV$ for fast GABA receptors. The fraction of open channels, $[O]$, is calculated according to the equation

$$\frac{d[O]}{dt} = \alpha(1-[O])[T]-\beta[O]$$

For cholinergic synapses transmitter concentration, $[T]$, is given by

$$[T] = A \cdot H(t_0+t_{max}-t) \cdot H(t-t_0)$$

and for GABAergic synapses

$$[T] = \frac{1}{1 + \exp(-(V - V_0)/\sigma)},$$

where $H$ is the Heaviside step function, $t_0$ is the time of receptor activation, $A = 0.5, t_{max} = 0.3ms, V_0 = -20mV$, and $\sigma = 1.5$. The rate constants were given $\alpha = 10ms^{-1}$ and $\beta = 0.2ms^{-1}$ for fast

$$GABA_A$$

synapses, and $\alpha = 1ms^{-1}$ and $\beta = 0.2ms^{-1}$ for cholinergic synapses.

The slow inhibitory synaptic current is given by the following equations:

$$I_{\text{slow-inh}} = g_{\text{slow-inh}} \frac{[G]^4}{[G]^4 + K}(V - E_k)$$

,

$$\frac{d[R]}{dt} = r_1(1 - [R])[T] - r_2[R]$$

,

$$\frac{d[G]}{dt} = r_3[R] - r_4[G]$$

where $[R]$ is the fraction of activated receptors, $[G]$ is the concentration of G proteins, $E_K = -95$ mV is the potassium reversal potential. The rate constants were $r_1 = 0.5$ mM$^{-1}$ms$^{-1}$, $r_2 = 0.0013$ ms$^{-1}$, $r_3 = 0.1$ ms$^{-1}$, $r_4 = 0.033$ ms$^{-1}$, and $K = 100$ $\mu$M$^4$. The peak synaptic conductances were set to $g_{GABA_A} = 0.02\mu S$ between LNs, $g_{GABA_A} = 0.015\mu S$ from LNs to PNs, $g_{ACh} = 0.3\mu S$ from PNs to LNs and $g$slow-inh $= 0.02$ $\mu$ from LNs to PNs.

For simulating the larger network with Gas Sensor Array Drift Dataset as input, the peak synaptic conductances were as follows: $g_{GABA_A} = 0.024\mu S$ between LNs, $g_{GABA_A} = 0.019\mu S$ from LNs to PNs and $g_{ACh} = 0.075\mu S$ from PNs to LNs.

### Plasticity

A simple phenomenological model of synaptic facilitation was used to model inhibitory plasticity (from LN to LN and LN to PN). Specifically, the inhibitory network underwent presynaptic facilitation when presented with a rewarded odor and postsynaptic facilitation when presented with a habituated odor during learning (Chen et al., 2015). Presynaptic (associated with reward) facilitation was assumed to depend on the octopamine receptors AmOA1 expressed in inhibitory LNs in the honey bee AL (Sinakevitch et al., 2013), (Sinakevitch et al., 2011) and it was based on the spiking events of the presynaptic neurons. In contrast, postsynaptic facilitation (associated with odor habituation) was based solely on the spiking event of the postsynaptic neuron (Sudhakaran et al., 2012), (Das et al., 2011).

To model facilitation, the maximum synaptic conductance is multiplied by a facilitation variable, $F$. $F$ is updated each time there is a spike:

$$Ft + 1 = F_t + dF_{pre} + dF_{post}$$

where $dF$ is facilitation rate ($dF_{pre} = 0.15$ and $dF_{post} = 0$ for presynaptic facilitation and $dF_{pre} = 0$ and $dF_{post} = 0.15$ for postsynaptic facilitation). $F_t$ is the value of the facilitation variable just before the spike. In the absence of any spiking events, $F$ had an exponential decay according to the following equation

$$F_t = 1 + (F_{t-1} - 1) * \exp\frac{-((t-1) - t_i)}{\tau}$$

In the above equation, $\tau = 30$s is the time constant of the decay; $t_i$ is the time of the $i^{th}$ spiking event; $F_t$ corresponds to the value of F at time $t$ with the initial value $F_0 = 1$. The time constant of decay is the same for pre and postsynaptic plasticity. The updated synaptic weights at the end of the training period (30 presentations, 60,000 ms) were frozen and used as the synaptic weights in the testing phase.

**Table 1** Connection probabilities between different neuron groups in the AL.

| Source Unit | Target Unit | Connection Probability |
|---|---|---|
| All PNs | All PNs | 0 |
| | Unipolar LNs | 0.4 |
| | Multipolar LNs | 0.4 |
| Unipolar LNs | PNs from same glomerulus | 0 |
| | PNs from different glomerulus | 0.5 |
| | Unipolar LNs from same glomerulus | 0 |
| | Unipolar LNs from different glomerulus | 0.4 |
| | Multipolar LNs | 0.3 |
| Multipolar LNs | All PNs | 0.3 |
| | Mutlipolar LNs | 0.1 |
| | Unipolar LNs | 0.4 |

### Network Geometry

The AL network included 100 PNs and 280 LNs. All these PNs and LNs were organised into 20 glomeruli, with each glomerulus containing 12 LNs (unipolar LNs) and 5 PNs. In addition to these, there are 40 LNs that contribute to global inhibition (multipolar LNs). The connections between each group of neurons were generated randomly based on the probabilities described in Table 1

In order to accommodate the ML-dataset inputs to the model, in some simulations we increased the number of neurons to 400 PNs and 1120 LNs. The larger network has random connectivity between LNs, LNs to PNs and PNs to LNs with a probability of 0.125. There were no connections between the different PNs.

### Odor Stimulation

To model odor stimulation, a fraction of LNs and PNs was activated by a current pulse with a rise time constant of 66.7ms and a decay time constant of 200ms for a period of 500ms as can be seen in Figure 2F. In addition, small amplitude current in the form of Gaussian Noise was added to each cell, to ensure random and independent membrane potential fluctuations. Each odor was modeled as a mixture of chemical components, with each pure chemical defined as a percept - the smallest unit of odor input. Each percept activated a group of "virtual ORNs", which in turn activate a certain group of PNs and LNs. Thus, each odor, consisting of multiple percepts (eg., 3 in the case of A,B odors and 6 in case of PH,PP odors), activated different overlapping groups of PNs and LNs. For example, for simulations shown in (Figure 3), the odor classes (A and B) were defined based on which 3 out of the 7 percepts were active for a given class. One odor class (A) was associated with reward and another one (B) with no reinforcement, with an overlap of 2 percepts. Individual odors within a class were defined by changing the level of LN/PN activation triggered by percepts.

In some simulations we modeled "PH-PP" odors which were created from the chemical compositions of two varieties of snapdragon odor blends (flowers) – PH (Pale Hybrid) and PP (Potomac Pink) as used in (Locatelli et al., 2016). Since there are 6 chemical components present in each odor, each chemical component was assigned a percept and the level of activation of each percept (width of the gaussian centered around the center of the percept in Fig5A) was calculated based on the proportion of that chemical component present in the natural odor blend. The $7^{th}$ percept remained inactive for all odors. For example, in the odor PH1, Oci chemical is 37.2% of the mixture. Hence, the standard deviation, which determines the width for the Gaussian, for percept 1 is set to 0.372 (Figure 5A).

Finally, we also used odor classes derived from the UCI Gas sensor dataset (Vergara et al., 2012). This dataset consists of data for 6 odors, obtained from 16 metal oxide sensors. Each odor consisted of 16 percepts, which activated a certain group of PNs and LNs. The first 200 out of 400 PNs and first 560 LNs out of 1120 LNs were provided with inputs, whereas the second half of PNs and LNs received no odor related inputs. This was done in order to maintain the E/I balance in the larger network. The first half of PNs and LNs were provided with odor pulses, created from 3 features of the data from each metal oxide sensor - $\Delta R$, $ema\_max_{\alpha=0.001}$ and $ema\_min_{\alpha=0.001}$ (Vergara et al., 2012). From these 3 features, we created an odor pulse with a rise time constant proportional to $1/ema\_max_{\alpha=0.001}$, decay time constant proportional to $1/ema\_min_{\alpha=0.001}$ and the maximum height of the pulse proportional to $\Delta R$. Of the 6 odors, 3 were associated with reward and 3 were associated with no reinforcement.

### Machine Learning Model - GCN model

In the last section of this paper, we applied a Graph Convolutional Network (GCN), a type of Graph Neural Network (GNN), to simulate AL training using machine learning approach. GCN is an approach for semi-supervised learning on graph structured data. It is convolutional in nature, because filter parameters are typically shared over all locations in the graph. GCN is a generalized version of CNNs which operate directly on arbitrarily structured graphs (Duvenaud et al., 2015). In (Kipf and Welling, 2016), the choice of convolutional architecture is motivated via a localized first-order approximation of spectral graph convolutions.

For the GCN model, the goal is to learn a function of signals/features on a graph which takes as input:

- A feature description $x_i$ for every node $i$; summarized in a N×D feature matrix $X$ (N: number of nodes, D: number of input features)
- A representative description of the graph structure in matrix form; typically in the form of an adjacency matrix $A$ (or some function thereof) and produces a node-level output $Z$ (an N×F feature matrix, where F is the number of output features per node). Graph-level outputs can be modeled by introducing some form of pooling operation. (Duvenaud et al., 2015)

Every neural network layer can then be written as a non-linear function $H(l+1) = f(H(l), A)$ with $H(0) = X$ and $H(L) = Z$, $L$ being the number of layers. The specific models then differ only in how $f$ is chosen and parameterized.

The UCI Gas Sensor Array Drift Dataset was taken as the input to the GCN, which consists of 2 parts, i.e, the graph convolutional layers and the fully connected layers. This dataset contains data for 6 different odors from 16 metal oxide sensors, with 8 features extracted from each sensor response, as described above. Thus, each odor is made of 128 features from 16 sensors. The data was normalized by subtracting the mean and dividing by standard deviation for each sensor value over the training and test sets. The GCN model consists of 16 (sensor) + 1 (reward/habituation) nodes and 2 layers of graph convolution. The output of the GCN is converted to a vector and sent to 2 fully connected layers for the final classification. The 6 odors from the dataset are split into 2 groups, one group associated with reward and one group associated with habituation. The task being performed by the model is to determine which group the given odor belongs to. The equation of this GCN model is, then, $H(l+1) = h(A \cdot H(l) \cdot W(l))$, where $l$ is the current layer and $A$ is the graph adjacency matrix with all connections between nodes belonging to sensors set to 1. The reward/habituation nodes' connections to the main 16 node graph was set during training as follows: if a rewarded odor was presented to the model during training, the connections between the reward and sensor nodes was set to 1. Conversely, if a habituated odor was presented to the model during training, the connection between reward node and sensor nodes was set to -1.

The model was trained via backpropagation, with the matrix W and the fully connected head of the model being updated during training. The cross entropy loss was optimized with the Adam Optimizer with a learning rate of 0.01 for 20 epochs. The graph convolution part of the model takes in 8 features from the 16 sensors and transform them into 4 abstract ones at the second layer. These abstract features encode the relationships between the different sensor features during training, similar to the AL which learn the relationships between the different odor percepts during training. Hence, we can say that the GCN model performs input preprocessing by learning the relationships between input features similar to the AL in honeybees. The first layer of the GCN can be thought of as analogous to the untrained AL network and the second layer can be thought of as the trained AL network.

## Analysis

### Euclidean Distance

Euclidean Distance (ED) was calculated based on the normalized binned spike counts across all the PNs for a single trial during the period of stimulation, with a bin size of 100ms. Normalization of the 100 dimensional vector for each time bin was done by dividing by the norm of the vector. The ED was calculated for each time bin and each trial and averaged over them.

### PCA trajectory

The response trajectory of the entire PN population to a specific input was constructed by binning the spike trains in 40ms bins. For visualisation, the 100 dimensional space of PN responses was reduced to 2D/3D space using Principal Component Analysis (PCA).

### Uniqueness index calculation

The uniqueness index for each glomerulus/percept (unit) was calculated as follows:

$$\text{UI} = \frac{(act\_unit^{\text{rew}}_{\text{naive}} - act\_unit^{\text{hab}}_{\text{naive}})}{act\_unit^{\text{hab}}_{\text{naive}}}$$

and the change in activity of the neurons:

$$\text{Change in activity} = \frac{act\_unit^{\text{rew}}_{\text{diff}} - act\_unit^{\text{rew}}_{\text{naive}}}{act\_unit^{\text{rew}}_{\text{naive}}}$$

where $act\_unit^{\text{rew}}_{\text{naive}}$ denotes the activation of the glomerulus by the rewarded odor and $act\_unit^{\text{hab}}_{\text{naive}}$ denotes the activation by the habituated odor before training (in the naive condition) and $act\_unit^{\text{rew}}_{\text{diff}}$ denotes the activation of the glomerulus by the rewarded odor after differential conditioning. A high positive Uniqueness index (UI) indicates that the given glomerulus is uniquely activated by the rewarded odors and a UI close to 0 would indicate the glomerulus is activated to a common extent by both rewarded and habituated odors. This method of calculating the UI was used for the biophysical models.

The uniqueness index for the GCN model was calculated based on the average activation (average of 8 feature values) for each sensor in GCN layer 1 for rewarded and habituated odors. The UI for the GCN is:

$$\text{UI} = \frac{act\_unit^{\text{rew}}_{\text{L1}} - act\_unit^{\text{hab}}_{\text{L1}}}{act\_unit^{\text{hab}}_{\text{L1}}}$$

where $act\_unit^{\text{rew}}_{\text{L1}}$ corresponds to the average activation of the GCN unit for the rewarded odors in layer 1 and $act\_unit^{\text{hab}}_{\text{L1}}$ corresponds to the average activation of the GCN unit for the habituated odors in layer 1. The change in activity was calculated based on the difference in average activation of each sensor node between layer 1 and layer 2 of the GCN:

$$\text{Change in activity} = act\_unit^{\text{rew}}_{\text{L2}} - act\_unit^{\text{rew}}_{\text{L1}}$$

## 5 Acknowledgments

This work was supported by grants - NSF (2223839, 2323241), NIH (R01DC020892) and NSF/CIHR/DFG/FRQ/UKRI-MRC Next Generation Networks for Neuroscience Program (2014217).

## Declarations

- Funding - This work was supported by grants - NSF (2223839, 2323241), NIH (R01DC020892) and NSF/CIHR/DFG/FRQ/UKRI-MRC Next Generation Networks for Neuroscience Program (2014217).
- Competing interests - The authors declare no competing interests.
- Ethics approval and consent to participate - Not applicable.
- Consent for publication - All authors consent to the publication of this manuscript.
- Data availability - The data will be made available after publication.
- Materials availability - Not applicable.
- Code availability - The code is available on Dr Bazhenov's lab website.
- Author contributions - S.J., S.H., Z.W., M.B. and Y.C. conceived and designed the research. S.J., S.H., F.L. and Z.W. performed the experiments. S.J. and S.H. analyzed the data. S.J. and S.H. drafted the manuscript. S.J. prepared the figures. S.J., S.H., Z.W., F.L., B.S., Y.C., M.B. edited and revised the manuscript.
