# Supplementary material for: Plasticity in inhibitory networks improves pattern separation in early olfactory processing
Source: bioRxiv. 2025 Feb 20:2024.01.24.576675. Originally published 2024 Jan 24. Preprint. [Version 3] doi: 10.1101/2024.01.24.576675 (PMC10849730; doi:10.1101/2024.01.24.576675)
Supplement: Supplement 1 [file NIHPP2024.01.24.576675v3-supplement-1.pdf]

bioRxiv preprint doi: https://doi.org/10.1101/2024.01.24.576675; this version posted February 20, 2025. The copyright holder for this preprint (which was not certified by peer review) is the author/funder, who has granted bioRxiv a license to display the preprint in perpetuity. It is made available under aCC-BY 4.0 International license.

Supplementary figures

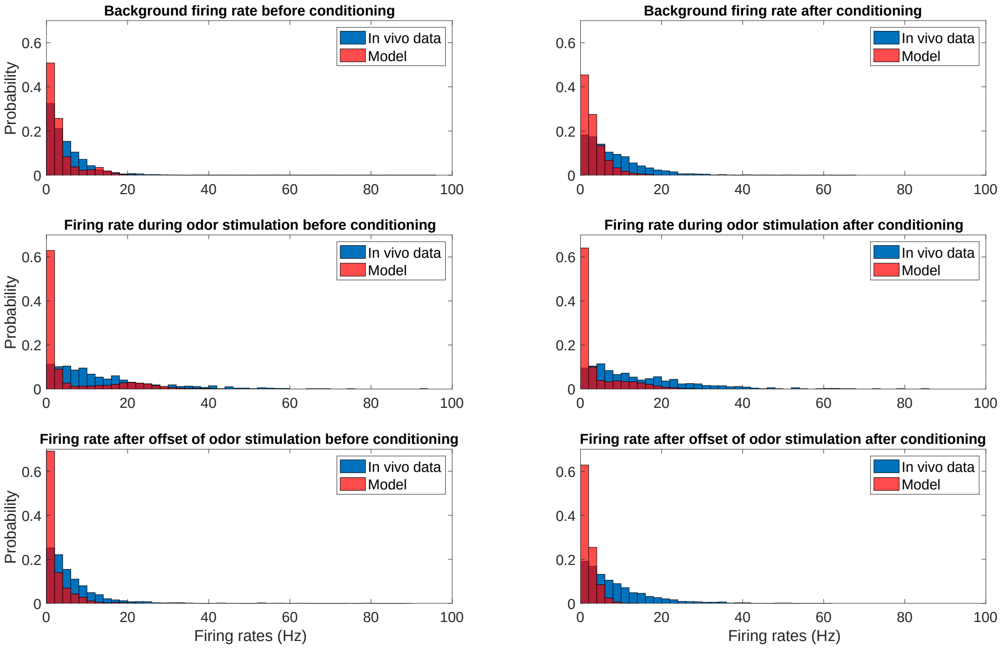

Fig. 1 Histograms of firing rates for in vivo and model PN responses for before and after conditioning. The three rows show firing rates calculated during different time periods. Row 1 shows firing rates for background activity. Row 2 shows firing rates calculated during 500ms of odor stimulation. Row 3 shows firing rates calculated 1s after the offset of the odor stimulation.

bioRxiv preprint doi: https://doi.org/10.1101/2024.01.24.576675; this version posted February 20, 2025. The copyright holder for this preprint (which was not certified by peer review) is the author/funder, who has granted bioRxiv a license to display the preprint in perpetuity. It is made available under aCC-BY 4.0 International license.

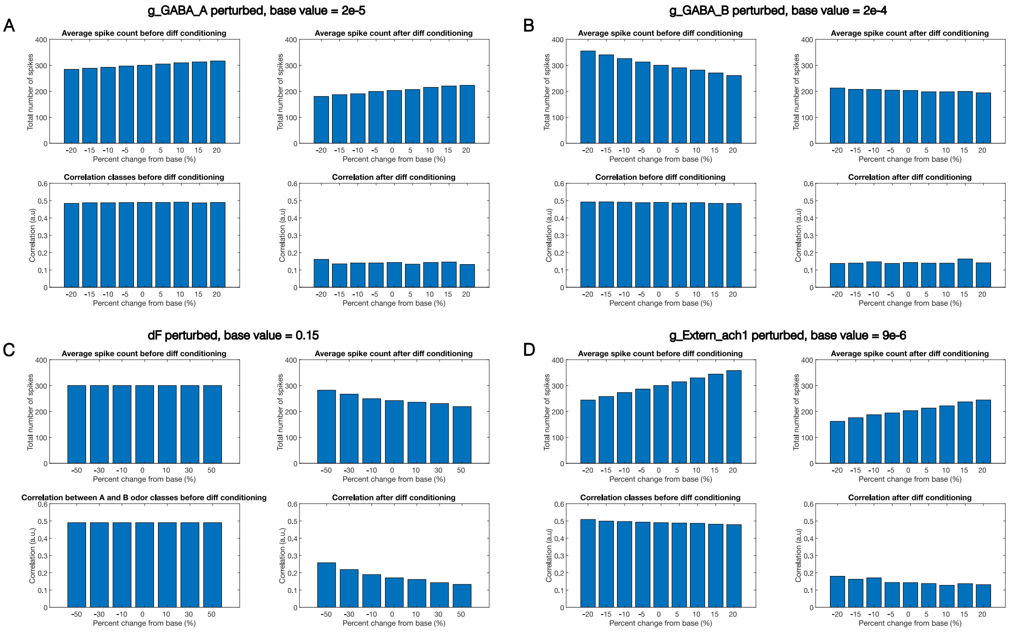

Fig. 2 Parameter sensitivity analysis demonstrates robustness of model behavior. (A-D) Each panel shows the effect of perturbing a different parameter: (A) fast GABA inhibition (g_GABA_A, base value = 2.0e-5), (B) slow GABA inhibition (g_GABA_B, base value = 2.0e-4), (C) facilitation rate (dF, base value = 1.5e-1), and (D) input synaptic conductance (g_Extern_ach1, base value = 9.0e-6). For each parameter, the top row shows the average spike count across all PNs before (left) and after (right) differential conditioning, while the bottom row shows the correlation between odor classes A and B before (left) and after (right) differential conditioning. The x-axis represents the percent change from the baseline parameter value. Despite variations in absolute spike counts, the key finding of reduced correlation between odor representations after differential conditioning remains robust across parameter perturbations, indicating that the model's core behavior does not depend on precise parameter tuning.

bioRxiv preprint doi: https://doi.org/10.1101/2024.01.24.576675; this version posted February 20, 2025. The copyright holder for this preprint (which was not certified by peer review) is the author/funder, who has granted bioRxiv a license to display the preprint in perpetuity. It is made available under aCC-BY 4.0 International license.

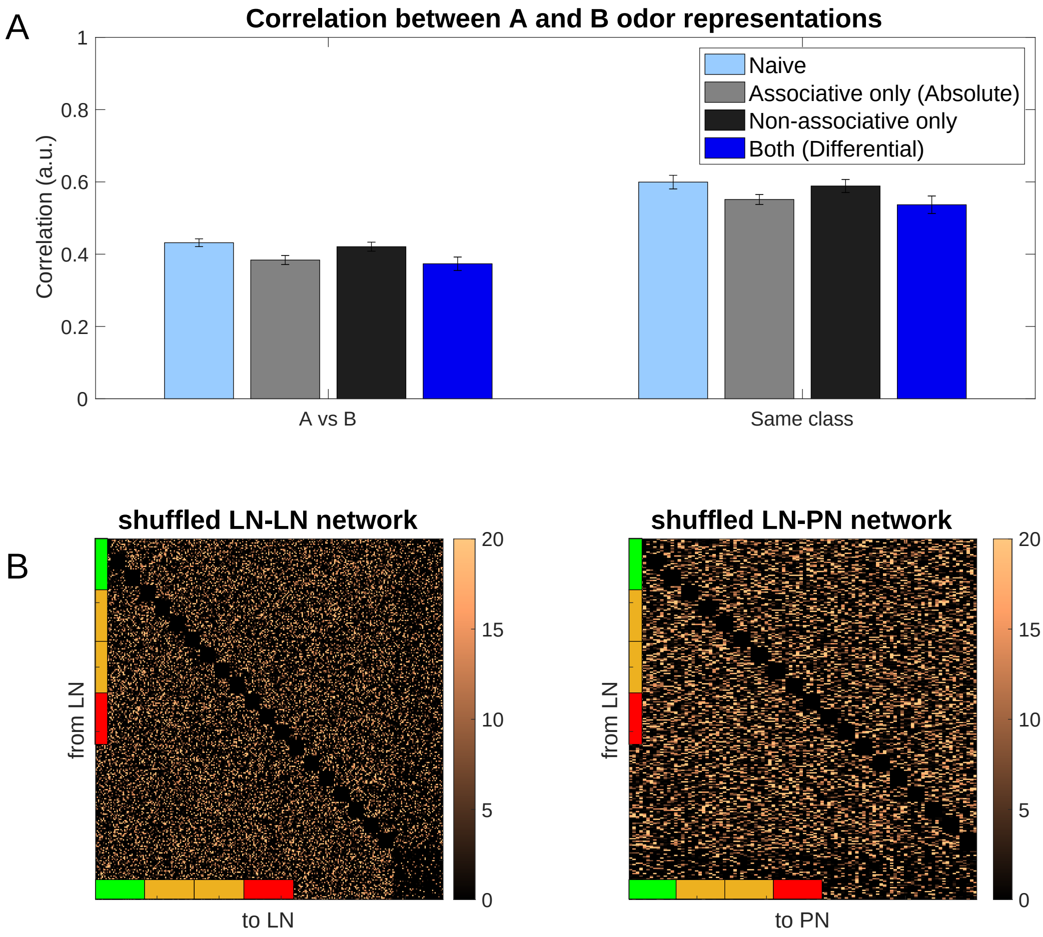

Fig. 3 Analysis of network connectivity structure and odor discrimination. A) Correlation between odor representations comparing naive, associative-only, non-associative-only, and combined (differential) conditioning for odors between different classes (left) and for odors within the same class (right). B) Connectivity matrices showing shuffled LN-LN and LN-PN networks after differential conditioning. When the structured connectivity patterns are disrupted through shuffling while maintaining the same total inhibitory strength, the enhanced odor discrimination is lost, demonstrating that the specific spatial organization of inhibitory connections, not just increased inhibition, is essential for improved odor discrimination. Side bars indicate percept identities (green: unique to rewarded odor, orange: shared, red: unique to habituated odor). Color bars show relative synaptic weights.
