# Supplementary material for: Plasticity in inhibitory networks improves pattern separation in early olfactory processing
Source: bioRxiv. 2024 Jan 24:2024.01.24.576675. Preprint. [Version 1] doi: 10.1101/2024.01.24.576675 (PMC10849730; doi:10.1101/2024.01.24.576675)
Supplement: Supplementary file 1 [file media-1.docx]

Supplementary Figures

**Supplementary Fig. 1: Raster plots for PN responses to the rewarded (A1) and habituated (B1) odors for all 4 cases - Naive, Associative plasticity only, Non-associative plasticity only, both associative and non-associative plasticity switched on during training**. A) Raster plots for PN responses to odor A1. The green bar at the bottom  denotes the time of odor stimulation by the rewarded odor. B) Raster plots for PN responses to odor B1. The red bar at the bottom  denotes the time of odor stimulation by the habituated odor. Here, we can see that the associative plasticity reduces the firing of PNs in the overlap percept (PNs denoted by orange bars to the right), while the non-associative plasticity increases the firing of PNs in the rewarded only percept (PNs denoted by green bar to the right). A combined effect of the associative and non-associative plasticity gives rise to the contrast enhancement phenomenon. The green, orange and red bars on the right side of the plot denote the rewarded, overlapping and habituated percepts respectively.

**Supplementary Fig. 2: Distance between representations of odors belonging to the same class in the floral dataset (PH - rewarded and PP - habituated odors).** For both classes, the distance between individual odors within a class increases after differential conditioning compared to naive and absolute conditioning case. This is similar to the experimentally observed change in the correlation of representations.
